# Scoparone-Induced Phase Separation of ZDHHC12 Drives Ferroptosis via ATG12 S-palmitoylation and Ferritinophagy in Liver Fibrosis

**DOI:** 10.34133/research.1450

**Published:** 2026-09-30

**Authors:** Yuyao Wei, Junli Xu, Jumei Tang, Jiaxin Zuo, Kaiwen Cheng, Chunbo Jiang, Yanshuang Zhuang, Mei Guo, Zili Zhang

**Affiliations:** ^1^ Jiangsu Key Laboratory for Pharmacology and Safety Research of Chinese Materia Medica, Nanjing University of Chinese Medicine, Nanjing 210023, China.; ^2^ School of Pharmacy, Nanjing University of Chinese Medicine, Nanjing 210023, China.; ^3^ TCM Nursing Intervention Key Laboratory of Chronic Disease, Nanjing University of Chinese Medicine, Nanjing 210023, China.; ^4^ Department of Nephrology, Suzhou Traditional Chinese Medicine Hospital Affiliated to Nanjing University of Chinese Medicine, Suzhou 210023, China.; ^5^ Department of Science and Technology, Taizhou Affiliated Hospital of Nanjing University of Chinese Medicine, Taizhou 225300, China.

## Abstract

Activated hepatic stellate cells (HSCs) are central effectors of liver fibrogenesis, and their selective elimination is critical for fibrosis regression. Although ferroptotic clearance of HSCs has emerged as a promising antifibrotic strategy, the upstream mechanisms coupling metabolic remodeling to ferroptosis susceptibility remain unclear. This study investigated whether scoparone (SCO) suppresses liver fibrosis through zinc finger DHHC-type palmitoyltransferase 12 (ZDHHC12) condensate formation, leading to enhanced autophagy-related protein 12 (ATG12) S-palmitoylation, induction of autophagy, and increased ferroptosis susceptibility in HSCs. We found that SCO restrained cell proliferation, migration, and fibrogenic activation while inducing iron overload, lipid peroxidation, glutathione depletion, and ferroptosis-like mitochondrial remodeling. Metabolomic and S-palmitoylation analyses linked these effects to increased palmitic acid levels and enhanced global protein S-palmitoylation. ZDHHC12 emerged as a SCO-responsive palmitoyltransferase associated with autophagy activation, ferritin turnover, ferroptosis induction, and repression of fibrogenic markers. Mechanistically, SCO promoted the formation of liquid-like ZDHHC12 condensates, which enhanced its interaction with ATG12 and facilitated ATG12 S-palmitoylation at the conserved Cys134 residue. This modification was associated with increased ATG12 stability through reduced ubiquitin-dependent proteasomal degradation, thereby promoting autophagy activation and increasing ferroptosis susceptibility in HSCs. Mutation of ATG12 at Cys134 attenuated these effects and diminished HSC ferroptosis. In cholestatic and toxic models of liver fibrosis, SCO alleviated liver injury and collagen deposition, whereas HSC-targeted silencing of ZDHHC12 or ATG12 attenuated these protective effects, supporting biological consistency in vivo. Integrated bioinformatic and clinical cohort analyses suggested that ZDHHC12 expression positively correlated with ATG12 expression and was associated with fibrosis progression in patients with liver fibrosis. Collectively, these findings support a ZDHHC12-dependent ATG12 S-palmitoylation pathway linking lipid metabolic remodeling to autophagy-mediated ferroptosis in HSCs and suggest phase-regulated S-palmitoylation as a potential antifibrotic target.

## Introduction

Liver fibrosis is driven largely by the persistent activation of hepatic stellate cells (HSCs), which acquire proliferative and contractile properties and become a major source of extracellular matrix in chronically injured livers [1,2]. Selective elimination of activated HSCs is therefore considered an important strategy for promoting fibrosis regression [3]. Ferroptosis, an iron-dependent form of regulated cell death characterized by lipid peroxidation and redox imbalance, has emerged as a potential mechanism for clearing activated HSCs [4,5]. Given that ferroptotic sensitivity is closely linked to intracellular iron availability, regulatory mechanisms that control iron storage and mobilization modulate ferroptosis. Ferritinophagy is a selective form of autophagy that degrades ferritin and releases redox-active iron, thereby linking autophagic activity to ferroptotic susceptibility [6]. Our previous work showed that ferritinophagy promotes iron mobilization and ferroptotic death in HSCs, supporting the relevance of this pathway in liver fibrosis [7]. However, how upstream metabolic signals regulate the autophagy pathway to initiate ferritinophagy and ferroptosis remains poorly understood.

S-palmitoylation is a reversible lipid modification in which a palmitoyl group is thioester-linked to a cysteine residue [8]. This modification is dynamically regulated by ZDHHC family palmitoyl acyltransferases and acyl protein thioesterases, allowing proteins to respond rapidly to metabolic and stress signals [9]. By increasing local hydrophobicity and membrane affinity, S-palmitoylation can reshape protein trafficking, subcellular distribution, and enzymatic activity [10]. Moreover, it may regulate protein stability by altering substrate accessibility to the ubiquitination machinery, thereby modulating ubiquitin-dependent proteasomal degradation [11,12]. Emerging evidence indicates that S-palmitoylation influences ferroptotic susceptibility by modifying the stability and function of proteins involved in lipid redox homeostasis [13,14]. However, previous studies have focused mainly on antioxidant pathways in cancer cells. Whether S-palmitoylation regulates the autophagy pathway involved in ferroptosis in activated HSCs remains unknown. Defining this mechanism may help connect metabolic signaling to the ferroptotic clearance of activated HSCs and provide a potential molecular target for antifibrotic intervention.

Liquid–liquid phase separation (LLPS) refers to a reversible process that organizes biomolecules into membrane-free condensates [15]. These condensates remain highly dynamic, allowing rapid molecular exchange with the surrounding environment while selectively concentrating proteins, substrates, enzymes, and regulatory factors into functional reaction hubs [16]. By increasing local reactant concentrations and prolonging contact times between enzymes and substrates, LLPS creates specialized microenvironments that can enhance substrate accessibility and catalytic efficiency [17]. Thus, LLPS provides spatial control over enzymatic reactions, including reversible posttranslational modifications that require coordinated interactions among enzymes, substrates, and metabolic donors [18]. This property raises the possibility that LLPS may regulate S-palmitoylation by locally enriching ZDHHC palmitoyltransferases and their substrates. Although phase separation has been implicated in autophagy and ferroptosis [19], whether palmitoyltransferases form functional condensates to promote S-palmitoylation remains unknown. Elucidating this mechanism may provide a framework for understanding LLPS-dependent spatial regulation of ZDHHC-mediated S-palmitoylation in ferroptosis.

Scoparone (SCO), chemically identified as 6,7-dimethoxycoumarin, is a naturally occurring coumarin derivative characterized by a benzopyrone scaffold bearing 2 methoxy groups [20]. It is one of the major bioactive constituents of *Artemisia scoparia*, a medicinal plant traditionally used for the treatment of hepatobiliary disorders [21]. Pharmacological studies have shown that SCO exerts hepatoprotective, antioxidant, anti-inflammatory, and metabolic regulatory activities in various experimental models of liver injury [22]. Our previous studies demonstrated that SCO suppresses HSC activation, reduces extracellular matrix deposition, and attenuates liver fibrosis, supporting its potential as an antifibrotic agent [23]. These findings provide the primary pharmacological rationale for selecting SCO in the present study. Notably, the metabolic regulatory properties of SCO suggest that it may reshape intracellular stress responses that influence cell-fate decisions. However, whether SCO promotes the elimination of activated HSCs by increasing ferroptotic susceptibility remains unclear. Clarifying this mechanism is essential for understanding the antifibrotic action of SCO.

In this study, we hypothesized that SCO promotes ferroptotic clearance of activated HSCs through an LLPS-dependent zinc finger DHHC-type palmitoyltransferase 12 (ZDHHC12)–autophagy-related protein 12 (ATG12) palmitoylation axis. Defining this pathway may provide mechanistic insight into the potential contribution of SCO-induced ZDHHC12 condensates to ATG12 S-palmitoylation, ferroptotic susceptibility, and fibrosis attenuation.

## Results

### SCO suppresses HSC activation by promoting ferroptosis

Given that HSC activation is a key event in liver fibrogenesis, we first sought to determine whether SCO can suppress HSC activation. 5-Ethynyl-2′-deoxyuridine (EdU) images and quantitative analyses indicated that SCO dose-dependently inhibited HSC proliferation in primary human HSCs (hHSCs) and HSC-LX2 cells. Silymarin (SM), included as a positive control, also reduced EdU incorporation (Fig. 1A and Figs. S1A and S2A). Moreover, western blot and quantitative real-time polymerase chain reaction (RT-qPCR) analyses showed that SCO decreased the expression of the HSC activation markers α-smooth muscle actin (α-SMA), collagen I, and fibronectin in a dose-dependent manner (Fig. 1B and C and Fig. S1B). Immunofluorescence images showed reduced α-SMA staining after SCO treatment (Fig. 1D). Cell Counting Kit-8 assays demonstrated that SCO did not substantially reduce overall cell viability at the concentrations used for the expression analyses, suggesting that the observed inhibitory effects were not attributable to nonspecific cytotoxicity (Fig. S2B). To evaluate the effect of SCO in a highly activated cellular context, HSC-LX2 cells were stimulated with transforming growth factor-β1 (TGF-β1) before SCO treatment. As expected, TGF-β1 increased the expression of *ACTA2*, *COL1A1*, and *FN1*, while these increases were attenuated by SCO (Fig. S2C). Furthermore, wound-healing assays revealed that SCO inhibited HSC migration (Fig. 1E and Fig. S2D). Cell cycle analysis showed that SCO increased the proportion of cells in S phase while decreasing the G2/M population (Fig. 1F and Fig. S2E). We next investigated whether ferroptotic signaling was associated with the inhibitory effects. SCO treatment increased intracellular iron, lipid reactive oxygen species (ROS), and malondialdehyde (MDA) levels while reducing glutathione (GSH) content in primary hHSCs and HSC-LX2 cells (Fig. 1G and Fig. S1C). Transmission electron microscopy revealed mitochondrial shrinkage following SCO treatment, consistent with ferroptotic morphology (Fig. 1H). In addition, western blot and RT-qPCR results indicated that SCO decreased the expression of the ferroptosis-suppressing factors glutathione peroxidase 4 (GPX4) and solute carrier family 7 member 11 (SLC7A11) and increased the expression of the ferroptosis-associated gene acyl-coenzyme A (CoA) synthetase long-chain family member 4 (ACSL4) (Fig. 1I). Prussian blue staining provided additional evidence of increased intracellular iron accumulation following SCO exposure (Fig. 1J). To assess whether ferroptotic signaling contributes functionally to the effects of SCO, cells were cotreated with the ferroptosis inhibitor ferrostatin-1 (Fer-1). Interestingly, Fer-1 attenuated SCO-induced iron accumulation, lipid ROS production, MDA elevation, and GSH depletion (Fig. 1K and Fig. S1D). SCO treatment inhibited GPX4 activity, whereas this suppression was reversed by cotreatment with either Fer-1 or liproxstatin-1 (Lip-1) (Fig. 1L). Consistently, Fer-1 alleviated the SCO-induced reduction in EdU incorporation (Fig. 1M and Figs. S1E and S2F) and restored the expression of HSC activation markers (Fig. 1N and O and Fig. S1F). Overall, these results suggest that SCO-induced suppression of HSC activation is associated with the induction of ferroptosis.

**Fig. 1.**
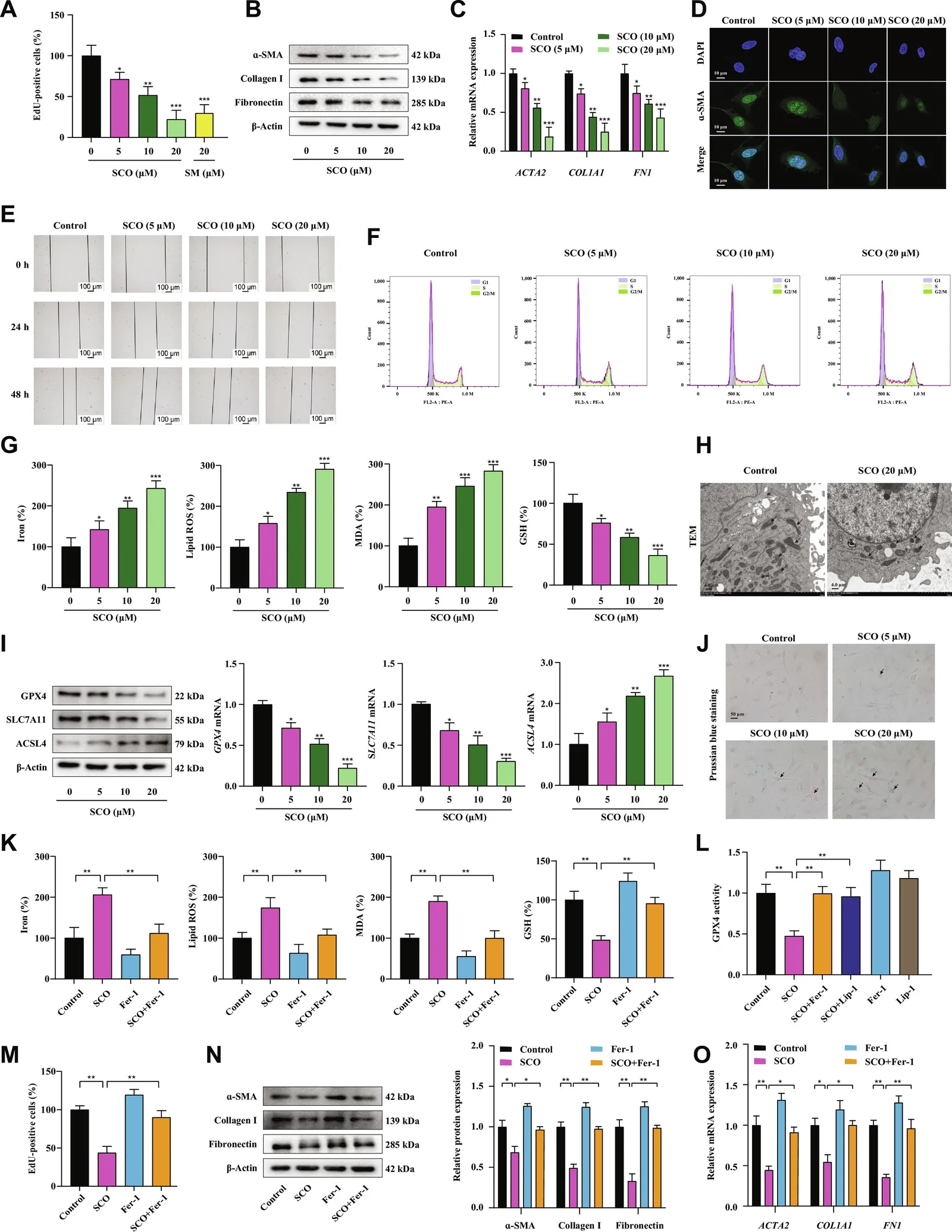
Scoparone (SCO) inhibits hepatic stellate cell (HSC) activation by inducing ferroptosis. HSC-LX2 cells were treated with SCO (5, 10, and 20 μM) or silymarin (SM, 20 μM) for 24 h. (A) Cell proliferation was assessed by the 5-Ethynyl-2′-deoxyuridine (EdU) assay (*n* = 3, ^*^P < 0.05, ^**^P < 0.01, ^***^P < 0.001). (B and C) The expression of α-smooth muscle actin (α-SMA), collagen I, and fibronectin was determined by western blot and quantitative real-time polymerase chain reaction (RT-qPCR) (*n* = 3, ^*^P < 0.05, ^**^P < 0.01, ^***^P < 0.001). (D) The staining of α-SMA was examined by immunofluorescence. Bar: 10 μm (*n* = 3). (E) Cell migration was assessed by the wound-healing assay. Bar: 100 μm (*n* = 3). (F) Cell cycle distribution was determined by flow cytometry (*n* = 3). (G) The intracellular iron, lipid reactive oxygen species (ROS), malondialdehyde (MDA), and glutathione (GSH) levels were examined by commercial assay kits (*n* = 3, ^*^P < 0.05, ^**^P < 0.01, ^***^P < 0.001). (H) Ferroptotic morphology was observed by transmission electron microscopy (TEM). Bar: 4 μm (*n* = 3). (I) The expression of glutathione peroxidase 4 (GPX4), solute carrier family 7 member 11 (SLC7A11), and acyl-coenzyme A synthetase long-chain family member 4 (ACSL4) was determined by western blot and RT-qPCR (*n* = 3, ^*^P < 0.05, ^**^P < 0.01, ^***^P < 0.001). (J) Iron deposition was examined by Prussian blue staining. Bar: 50 μm (*n* = 3). HSC-LX2 cells were treated with 20 μM SCO and 10 μM ferrostatin-1 (Fer-1) or 10 μM liproxstatin-1 (Lip-1) for 24 h. (K) The intracellular iron, lipid ROS, MDA, and GSH levels were examined by commercial assay kits (*n* = 3, ^**^P < 0.01). (L) GPX4 activity was measured using a GPX4 activity assay (*n* = 3, ^**^P < 0.01). (M) Cell proliferation was assessed by the EdU assay (*n* = 3, ^**^P < 0.01). (N and O) The expression of α-SMA, collagen I, and fibronectin was determined by western blot and RT-qPCR (*n* = 3, ^*^P < 0.05, ^**^P < 0.01). DAPI, 4′,6-diamidino-2-phenylindole.

### ZDHHC12-dependent S-palmitoylation contributes to SCO-induced ferroptosis in HSCs

Emerging studies have highlighted a tight linkage between ferroptosis and metabolic remodeling [24–26]. To delineate the upstream events underlying SCO-induced ferroptosis in HSCs, we performed untargeted metabolomic profiling to assess global metabolic alterations. Hierarchical clustering and Kyoto Encyclopedia of Genes and Genomes (KEGG) pathway enrichment analyses of differentially abundant metabolites revealed that fatty acid biosynthesis was among the most significantly enriched pathways following SCO exposure (Fig. 2A to C). To validate this observation, we quantified representative intermediates along the fatty acid synthesis cascade. Liquid chromatography–mass spectrometry (LC-MS) analysis demonstrated that SCO increased the abundance of several fatty acids, with palmitic acid (C16:0) exhibiting the most prominent accumulation compared with other acyl chain lengths (Fig. 2D). To determine whether SCO enhances de novo palmitic acid synthesis, we performed metabolic flux analysis using ^13^C-labeled substrates. Interestingly, SCO treatment increased ^13^C incorporation into palmitic acid (Fig. S3A). Sterol regulatory element-binding protein 1 (SREBP1) functions as a master lipogenic transcription factor that promotes the expression of key enzymes involved in de novo fatty acid synthesis, including acetyl-CoA carboxylase (ACC) and fatty acid synthase (FASN) [27]. Western blot and RT-qPCR results showed that SCO up-regulated the expression of SREBP1, ACC1, and FASN (Fig. S3B). Pharmacological inhibition of SREBP1 using fatostatin attenuated palmitic acid accumulation (Fig. S3C), suggesting that activation of the lipogenic program may contribute to SCO-induced expansion of the C16 fatty acid pool. Given that palmitic acid serves as the essential substrate for S-palmitoylation [28], we investigated whether SCO modulates this posttranslational modification to promote HSC ferroptosis. Acyl-biotin exchange (ABE) assays demonstrated a dose-dependent increase in global protein S-palmitoylation upon SCO treatment (Fig. 2E). Mass spectrometry analysis of palmitoylated peptides further supported a broad enhancement of protein S-palmitoylation under SCO exposure (Fig. 2F). To determine whether S-palmitoylation contributes to ferroptosis induction, we pharmacologically manipulated this modification using the palmitoylation inhibitor 2-bromohexadecanoic acid (2-BP) and the agonist palmostatin B (Palm B) [29]. As expected, 2-BP attenuated SCO-triggered cell death, whereas Palm B enhanced the cell death response induced by SCO (Fig. 2G). Consistently, 2-BP mitigated SCO-induced iron accumulation and MDA elevation, while Palm B amplified these ferroptotic features (Fig. S3D). Because protein S-palmitoylation is dynamically regulated by protein acyltransferases (PATs) and acyl-protein thioesterases [30], we systematically profiled the expression of these enzymes. Although modest transcriptional alterations were observed in several ZDHHC family members, ZDHHC12 exhibited the most robust and consistent up-regulation among the PATs and acyl-protein thioesterases examined (Fig. 2H and I). Western blot and RT-qPCR analyses confirmed dose-dependent elevation of ZDHHC12 expression following SCO treatment (Fig. 2J and Fig. S1G). To further investigate the signaling pathways potentially involved in SCO-induced ZDHHC12 expression and fatty acid metabolism, we examined several candidate pathways, including mechanistic target of rapamycin (mTOR) signaling, AMP-activated protein kinase (AMPK) signaling, and the transcriptional regulator peroxisome proliferative activated receptor gamma (PPARγ) [31]. The results indicated that SCO treatment increased the p-mTOR/mTOR ratio, whereas the p-AMPK/AMPK ratio and PPARγ protein levels were not substantially affected (Fig. S3E). Inhibition of mTOR signaling with rapamycin reversed SCO-induced palmitic acid accumulation and reduced ZDHHC12 expression (Fig. S3F). To assess whether ZDHHC12 mediates the palmitoylation-dependent ferroptotic response, we generated ZDHHC12-targeting short hairpin RNA (shRNA) knockdown (KD) constructs (Fig. S3G). ZDHHC12 KD attenuated SCO-induced ferroptotic phenotypes, including lipid ROS accumulation, GSH depletion, MDA elevation, iron overload, GPX4 suppression, and ACSL4 up-regulation (Fig. 2K and Fig. S3H). EdU assays revealed that ZDHHC12 KD reversed the SCO-induced suppression of HSC proliferation (Fig. 2L and Fig. S3I). In addition, ZDHHC12 KD abrogated the antifibrotic efficacy of SCO, as evidenced by restoration of α-SMA, collagen I, and fibronectin expression (Fig. 2M and N). Altogether, these findings suggest that ZDHHC12-dependent S-palmitoylation contributes to SCO-induced HSC ferroptosis.

**Fig. 2.**
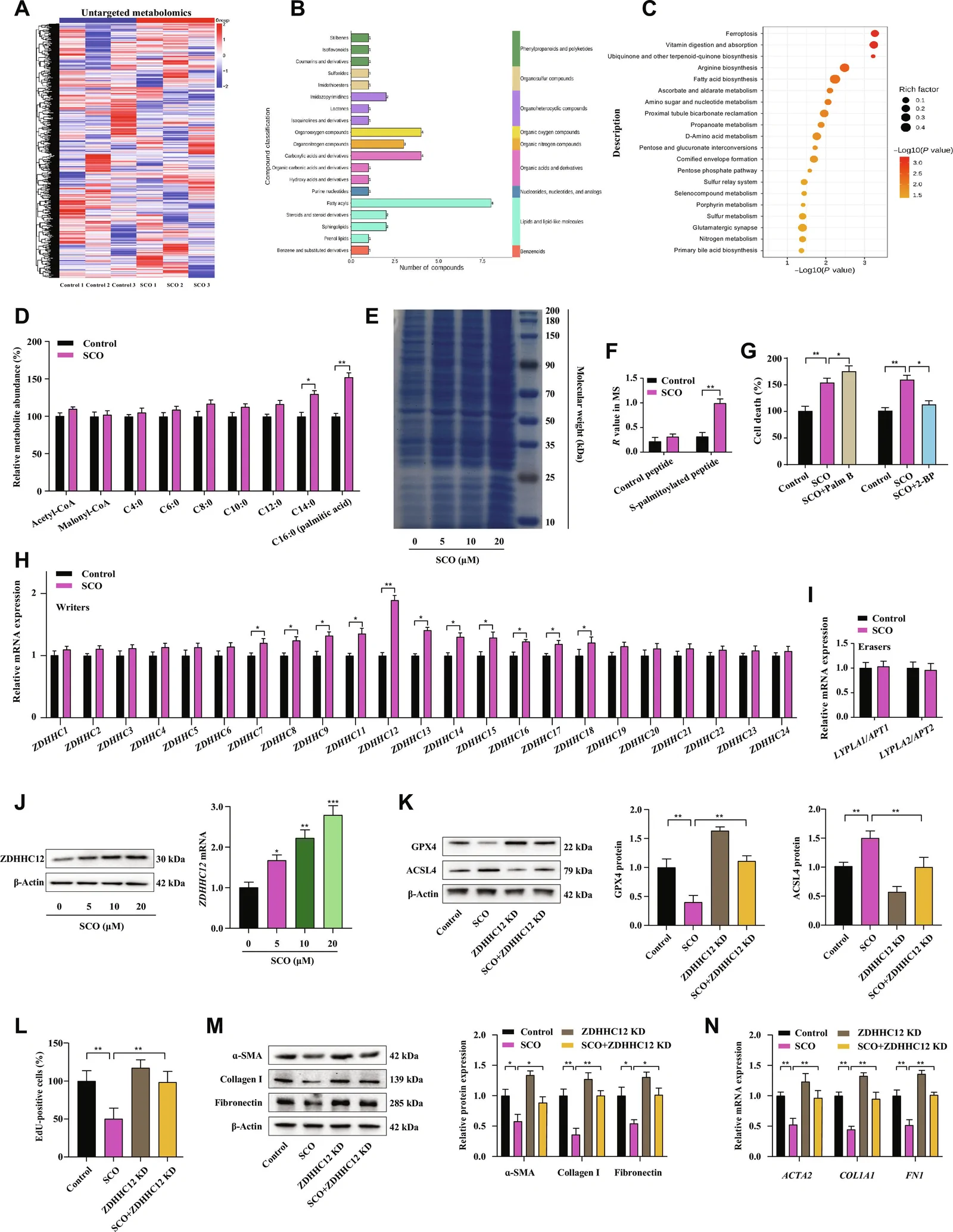
Scoparone (SCO) promotes hepatic stellate cell (HSC) ferroptosis by regulating zinc finger DHHC-type palmitoyltransferase 12 (ZDHHC12)-mediated S-palmitoylation. HSC-LX2 cells were exposed to SCO (20 μM) for 24 h. (A to C) Differential metabolites were identified by untargeted metabolomics (*n* = 3). (D) The relative abundance of fatty acid-related metabolites was detected by liquid chromatography–mass spectrometry (LC-MS) (*n* = 3, ^*^P < 0.05, ^**^P < 0.01). (E and F) Global protein S-palmitoylation levels were determined by sodium dodecyl sulfate-polyacrylamide gel electrophoresis (SDS-PAGE) and mass spectrometry (*n* = 3, ^**^P < 0.01). (G) HSC-LX2 cells were treated with 20 μM SCO and 10 μM 2-bromohexadecanoic acid (2-BP) or 10 μM palmostatin B (Palm B) for 24 h. Cell death was examined by propidium iodide (PI) staining (*n* = 3, ^*^P < 0.05, ^**^P < 0.01). (H and I) The expression levels of protein acyltransferases (PATs) and acyl-protein thioesterases (APTs) were measured by quantitative real-time polymerase chain reaction (RT-qPCR) (*n* = 3, ^*^P < 0.05, ^**^P < 0.01). (J) ZDHHC12 expression was determined by western blot and RT-qPCR (*n* = 3, ^*^P < 0.05, ^**^P < 0.01, ^***^P < 0.001). HSC-LX2 cells stably transfected with ZDHHC12 short hairpin RNA (shRNA) (knockdown [KD]) were exposed to SCO (20 μM) for 24 h. (K) The expression of glutathione peroxidase 4 (GPX4) and acyl-coenzyme A synthetase long-chain family member 4 (ACSL4) was determined by western blotting (*n* = 3, ^**^P < 0.01). (L) Cell proliferation was assessed by the 5-ethynyl-2′-deoxyuridine (EdU) assay (*n* = 3, ^**^P < 0.01). (M and N) The expression of α-smooth muscle actin (α-SMA), collagen I, and fibronectin was determined by western blot and RT-qPCR (*n* = 3, ^*^P < 0.05, ^**^P < 0.01).

### Autophagy activation is associated with S-palmitoylation-driven HSC ferroptosis in SCO-treated HSCs

To explore the molecular mechanism of S-palmitoylation regulating HSC ferroptosis, we performed transcriptome sequencing comparing the wild-type (WT), ZDHHC12 KD, SCO+WT, and SCO+ZDHHC12 KD groups. Comparison between WT and ZDHHC12 KD cells under basal conditions identified a subset of genes involved in protein processing in the endoplasmic reticulum, lipid modification-related pathway, and autophagy signaling pathway, suggesting that ZDHHC12 may participate in the regulation of protein quality control and lipid homeostasis in the absence of SCO (Fig. S4A and B). As illustrated by the heatmap (Fig. 3A) and circular heatmap (Fig. 3B), 528 differentially expressed genes were identified in the SCO+ZDHHC12 KD group relative to the SCO+WT group. Interestingly, gene ontology analysis and KEGG enrichment analysis revealed that the autophagy signaling pathway was among the significantly enriched pathways (Fig. 3C and Fig. S4C). To validate these transcriptomic observations, we examined key autophagy-related proteins. Western blot and RT-qPCR analyses showed that ZDHHC12 KD attenuated the SCO-induced formation of the ATG12–ATG5 conjugate, while ZDHHC12 overexpression (OE) increased its abundance (Fig. 3D and E). ATG12 is essential for autophagosome formation because its conjugation with ATG5 promotes microtubule-associated protein 1 light chain 3 (LC3) lipidation and autophagic membrane expansion [32]. Consistently, the LC3-II/LC3-I ratio was reduced in ZDHHC12 KD cells and elevated in ZDHHC12 OE cells under SCO treatment (Fig. 3F and G). In parallel, ZDHHC12 KD attenuated the SCO-induced reduction of p62 and ferritin heavy chain 1 (FTH1) protein levels, whereas ZDHHC12 OE further exacerbated this decrease (Fig. 3F and H and Fig. S4D). Representative immunofluorescence images showed that ZDHHC12 KD decreased the SCO-induced ATG12 and LC3 staining, whereas ZDHHC12 OE enhanced the effect of SCO (Fig. S4E). The red fluorescent protein–green fluorescent protein–LC3 (RFP-GFP-LC3) puncta results showed that ZDHHC12 KD reduced both autophagosome and autolysosome puncta, whereas ZDHHC12 OE increased their abundance in SCO-treated cells (Fig. 3I and L). Moreover, ZDHHC12 KD reduced LC3 puncta formation, while ZDHHC12 OE increased LC3 puncta abundance in SCO-treated cells (Fig. 3J and K). Transmission electron microscopy revealed increased numbers of double-membrane autophagic structures in SCO-treated cells compared with untreated controls (Fig. 3M). In addition, long-lived protein degradation assays demonstrated that ZDHHC12 KD attenuated SCO-induced autophagic degradation, while ZDHHC12 OE enhanced this process (Fig. S4F). Collectively, these data suggest that ZDHHC12-dependent S-palmitoylation is associated with enhanced autophagic activity during SCO-induced HSC ferroptosis.

**Fig. 3.**
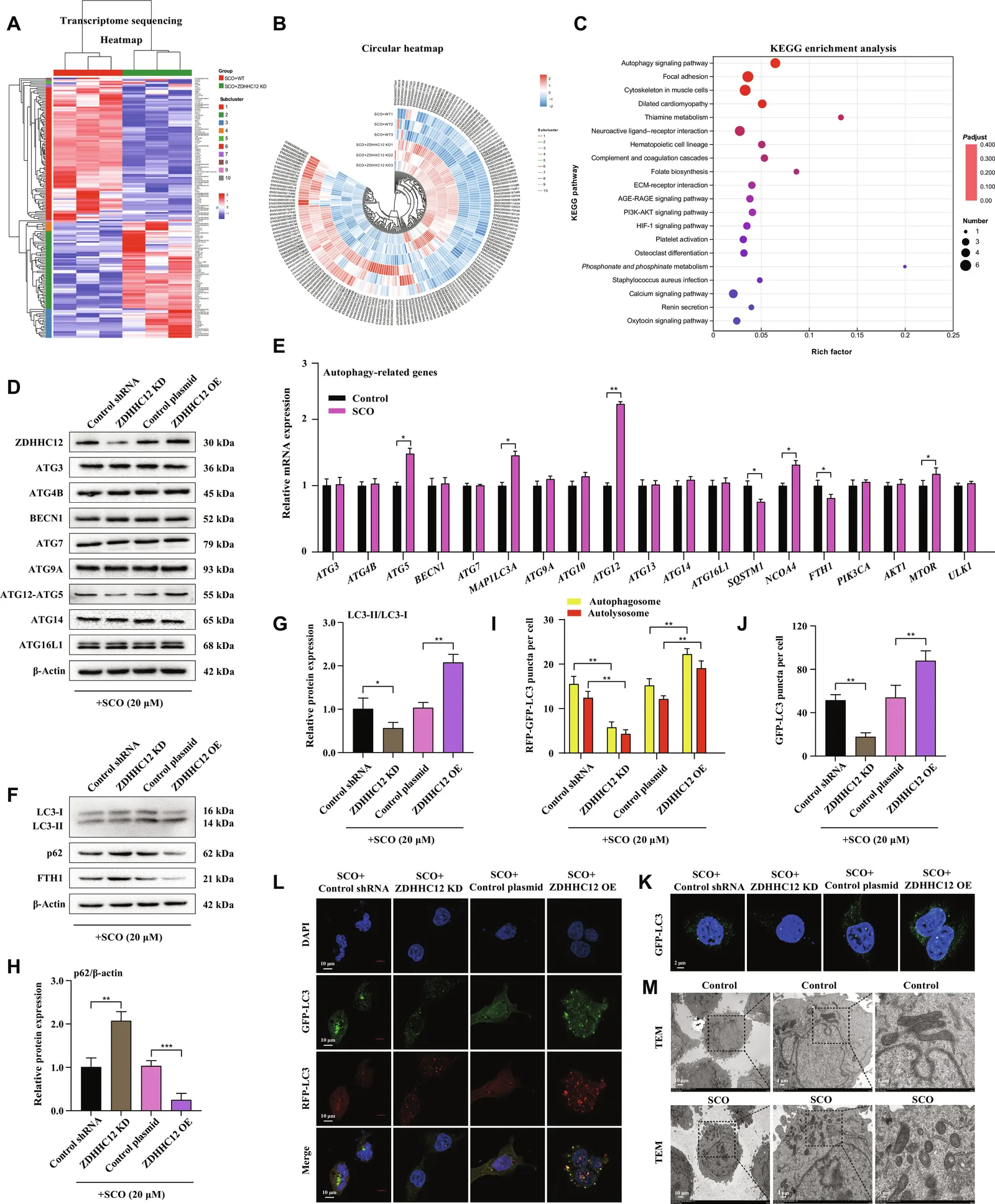
Autophagy activation is associated with zinc finger DHHC-type palmitoyltransferase 12 (ZDHHC12)-mediated hepatic stellate cell (HSC) ferroptosis in scoparone (SCO)-treated HSCs. HSC-LX2 cells stably transfected with ZDHHC12 short hairpin RNA (shRNA) (knockdown [KD]) or ZDHHC12 overexpression (OE) plasmid were exposed to SCO (20 μM) for 24 h. (A to C) Differentially expressed genes (DEGs) were identified by transcriptome sequencing (*n* = 3, |log_2_FC| > 1, adjusted *P* < 0.05). (D and E) The expression of autophagy-related proteins was determined by western blot and quantitative real-time polymerase chain reaction (RT-qPCR) (*n* = 3, ^*^P < 0.05, ^**^P < 0.01). (F to H) The expression of LC3-I/II and p62 was examined by western blotting (*n* = 3, ^*^P < 0.05, ^**^P < 0.01, ^***^P < 0.001). (I and L) Autophagic flux was assessed by red fluorescent protein–green fluorescent protein–LC3 (RFP-GFP-LC3) fluorescence puncta. Scale bar: 10 μm (*n* = 3, ^**^P < 0.01). (J and K) LC3 accumulation was evaluated by GFP-LC3 puncta. Scale bar: 2 μm (*n* = 3, ^**^P < 0.01). (M) Autophagosomes were detected by transmission electron microscopy. Scale bar: 10 μm (*n* = 3).

### ZDHHC12-mediated S-palmitoylation of ATG12 contributes to autophagy activation and SCO-mediated HSC ferroptosis

To identify potential substrates of ZDHHC12 in ferroptosis, Flag-tagged ZDHHC12 immunoprecipitation followed by mass spectrometry was performed in SCO-treated HSCs (Fig. 4A). Among the identified candidates, ATG12 was ranked within the top enriched proteins and exhibited specific association with Flag-ZDHHC12 under SCO exposure (Fig. 4A). Moreover, ABE assays revealed that SCO treatment led to a dose-dependent increase in ATG12 S-palmitoylation levels, whereas the other candidate proteins exhibited no marked alterations (Fig. 4B and Fig. S1H). Bioinformatic analysis using GPS-Palm identified Cys134 of ATG12 as a high-confidence palmitoylation site, and sequence alignment demonstrated high conservation of this region across different species (Fig. 4C). To further assess the functional relevance of Cys134, a site-directed mutant plasmid was generated in which cysteine 134 was substituted with alanine (ATG12 C134A). Western blot analysis suggested that ATG12 WT increased ATG12 protein levels under SCO treatment, whereas ATG12 C134A failed to produce a comparable increase (Fig. 4D and Fig. S5A). ABE assays showed that ATG12 WT enhanced SCO-induced ATG12 S-palmitoylation, whereas ATG12 C134A failed to enhance the effect of SCO (Fig. 4E and Fig. S5B). Consistently, ATG12 WT increased the LC3-II/LC3-I ratio and reduced p62 levels under SCO treatment, while ATG12 C134A failed to augment these changes (Fig. 4D and Fig. S5C). Furthermore, ATG12 WT increased intracellular iron levels, MDA production, and lipid ROS accumulation under SCO treatment, but ATG12 C134A did not produce an additional increase compared with SCO alone (Fig. 4F). ATG12 WT potentiated the SCO-mediated reduction in α-SMA, collagen I, and fibronectin expression, whereas ATG12 C134A failed to augment this suppressive effect (Fig. 4G). To further assess the functional relevance of ZDHHC12, rescue experiments were performed in ZDHHC12 KD cells by reexpression of ZDHHC12. Importantly, ZDHHC12 KD decreased ZDHHC12 and ATG12 protein levels, which were effectively restored by ZDHHC12 OE (Fig. 4H and Fig. S5D). ABE assays showed that ATG12 S-palmitoylation levels were reduced in ZDHHC12 KD cells and were restored upon reexpression of ZDHHC12 (Fig. 4I and J). Western blot analysis indicated that ZDHHC12 KD resulted in a decreased LC3-II/LC3-I ratio and increased p62 accumulation, and these alterations were restored upon ZDHHC12 reexpression (Fig. 4H and J). In addition, ZDHHC12 KD reduced intracellular iron levels, MDA production, and lipid ROS accumulation, and these changes were restored upon ZDHHC12 reexpression (Fig. 4K). ZDHHC12 deficiency was accompanied by elevated expression of α-SMA, collagen I, and fibronectin at the protein and messenger RNA (mRNA) levels, which were reversed following ZDHHC12 reexpression (Fig. 4L). Taken together, these results suggest that ZDHHC12 promotes ATG12 S-palmitoylation to induce autophagy and SCO-mediated HSC ferroptosis.

**Fig. 4.**
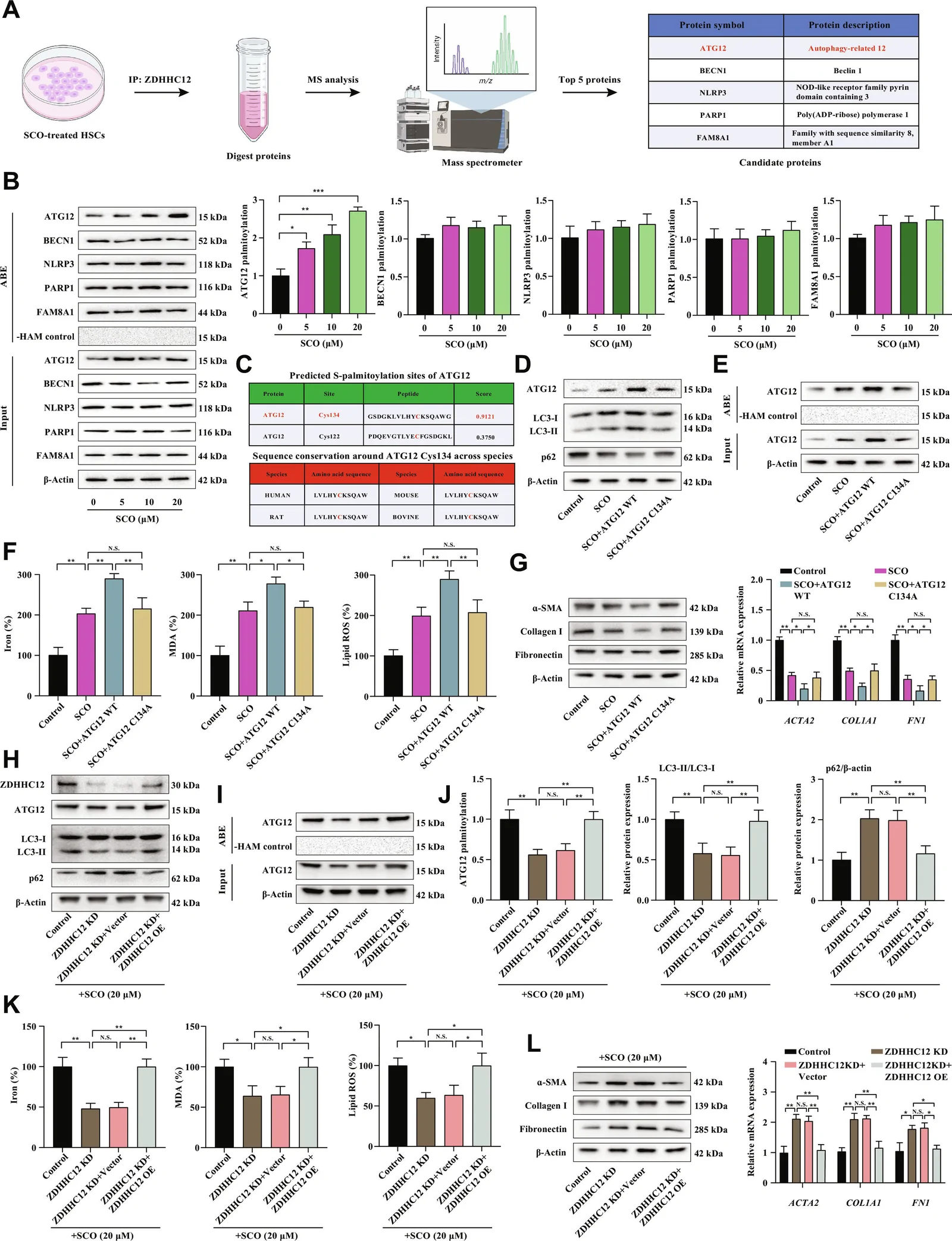
Zinc finger DHHC-type palmitoyltransferase 12 (ZDHHC12) enhances autophagy-related protein 12 (ATG12) S-palmitoylation to regulate ferroptosis in scoparone (SCO)-treated hepatic stellate cells (HSCs). HSC-LX2 cells were exposed to SCO (5, 10, and 20 μM) for 24 h. (A) A schematic workflow for identifying ZDHHC12-interacting proteins by immunoprecipitation–mass spectrometry. (B) The S-palmitoylation levels of ATG12, BECN1, NLRP3, PARP1, and FAM8A1 were determined by the acyl-biotin exchange assay (*n* = 3, ^*^P < 0.05, ^**^P < 0.01, ^***^P < 0.001). (C) S-palmitoylation sites of ATG12 were predicted by GPS-Palm. HSC-LX2 cells stably expressing ATG12 wild-type (WT) or ATG12 C134A mutant were exposed to SCO (20 μM) for 24 h. (D) The expression of ATG12, LC3-I/II, and p62 was determined by western blotting (*n* = 3). (E) The ATG12 S-palmitoylation was examined by the acyl-biotin exchange assay (*n* = 3). (F) The intracellular iron, lipid reactive oxygen species (ROS), and malondialdehyde (MDA) levels were detected by commercial assay kits (*n* = 3, ^*^P < 0.05, ^**^P < 0.01, N.S., not significant). (G) The expression of α-smooth muscle actin (α-SMA), collagen I, and fibronectin was determined by western blot and quantitative real-time polymerase chain reaction (RT-qPCR) (*n* = 3, ^*^P < 0.05, ^**^P < 0.01, N.S., not significant). Rescue experiments were performed by reexpressing ZDHHC12 in ZDHHC12 knockdown (KD) cells, followed by treatment with SCO (20 μM) for 24 h. (H and J) The expression of ZDHHC12, ATG12, LC3-I/II, and p62 was determined by western blotting (*n* = 3, ^**^P < 0.01, N.S., not significant). (I) The ATG12 S-palmitoylation was examined by the acyl-biotin exchange assay (*n* = 3). (K) The intracellular iron, lipid ROS, and MDA levels were detected by commercial assay kits (*n* = 3, ^*^P < 0.05, ^**^P < 0.01, N.S., not significant). (L) The expression of α-SMA, collagen I, and fibronectin was determined by western blot and RT-qPCR (*n* = 3, ^*^P < 0.05, ^**^P < 0.01, N.S., not significant).

### ZDHHC12 enhances ATG12 stability via S-palmitoylation in SCO-mediated HSC ferroptosis

To further delineate how ZDHHC12 regulates ATG12 S-palmitoylation, we first examined the potential interaction between the 2 proteins. Molecular docking analysis predicted a favorable binding conformation with a defined interaction interface between ATG12 and ZDHHC12 (Fig. 5A). Confocal microscopy results demonstrated that SCO treatment increased the colocalization of ATG12 and ZDHHC12 in a dose-dependent manner (Fig. 5B). Coimmunoprecipitation assays revealed that SCO treatment promoted the interaction between ZDHHC12 and ATG12 (Fig. 5C). Moreover, cycloheximide chase assays showed that SCO treatment prolonged the half-life of ATG12, whereas ZDHHC12 KD accelerated its degradation, suggesting a role for ZDHHC12 in maintaining ATG12 protein stability (Fig. 5D). Treatment with the proteasome inhibitor MG-132, but not the autophagy inhibitor 3-methyladenine, restored ATG12 protein levels in ZDHHC12-deficient cells (Fig. 5E), implicating the ubiquitin–proteasome system in ATG12 degradation. Consistently, ubiquitination assays demonstrated reduced ubiquitination levels of ATG12 following SCO treatment (Fig. 5F). Notably, accumulating evidence suggests that palmitoylation can regulate protein stability by altering substrate accessibility to the ubiquitination machinery, thereby modulating ubiquitination outcomes [11,12]. To examine whether Cys134 palmitoylation affects ATG12 ubiquitination, we generated HSC-LX2 cells stably expressing ATG12-WT or ATG12-Cys134A mutant. Interestingly, limited proteolysis assays demonstrated slower degradation kinetics and a prolonged proteolytic half-life for Cys134-palmitoylated ATG12 (Fig. S6A to C), while carrier-free nanomodulator analysis showed increased thermal stability (Fig. S6D). Moreover, SCO treatment enhanced the endogenous ATG12–ATG5 interaction, which was further strengthened by ATG12 WT but not by ATG12 C134A (Fig. S6E). Ubiquitination assays showed that ATG12-Cys134A displayed higher ubiquitination levels than ATG12-WT (Fig. S6F). Western blot analysis indicated that ATG12-Cys134A exhibited a lower protein level than ATG12-WT (Fig. S6F). To further determine whether ATG12 functions as a downstream effector of palmitoylation in SCO-induced ferroptosis, rescue experiments were performed by reexpressing ATG12 WT or ATG12 C134A in ATG12-deficient cells. Western blot analysis revealed that ATG12 KD reduced ATG12 protein levels, which were restored by ATG12 WT but not by ATG12 C134A under SCO treatment (Fig. 5G and I). ABE assays showed that ATG12 KD decreased ATG12 S-palmitoylation, while reexpression of ATG12 WT restored S-palmitoylation levels. In contrast, ATG12 C134A failed to rescue ATG12 S-palmitoylation (Fig. 5H and I). ATG12 KD reduced the LC3-II/LC3-I ratio and increased p62 protein levels, which was recovered by ATG12 WT but not by ATG12 C134A (Fig. 5G and J). Consistently, ATG12 KD reduced intracellular iron levels, MDA production, and lipid ROS accumulation. These decreases were reversed by reexpression of ATG12 WT, whereas ATG12 C134A exhibited no rescue effects (Fig. 5K and Fig. S1J). In parallel, ATG12 KD increased HSC proliferation and fibrogenic marker expression under SCO treatment. Reexpression of ATG12 WT attenuated these increases, whereas ATG12 C134A failed to produce a comparable rescue (Fig. 5L to N and Fig. S1K). Altogether, these findings indicate that ZDHHC12 induces ATG12 S-palmitoylation and enhances its stability in SCO-mediated HSC ferroptosis.

**Fig. 5.**
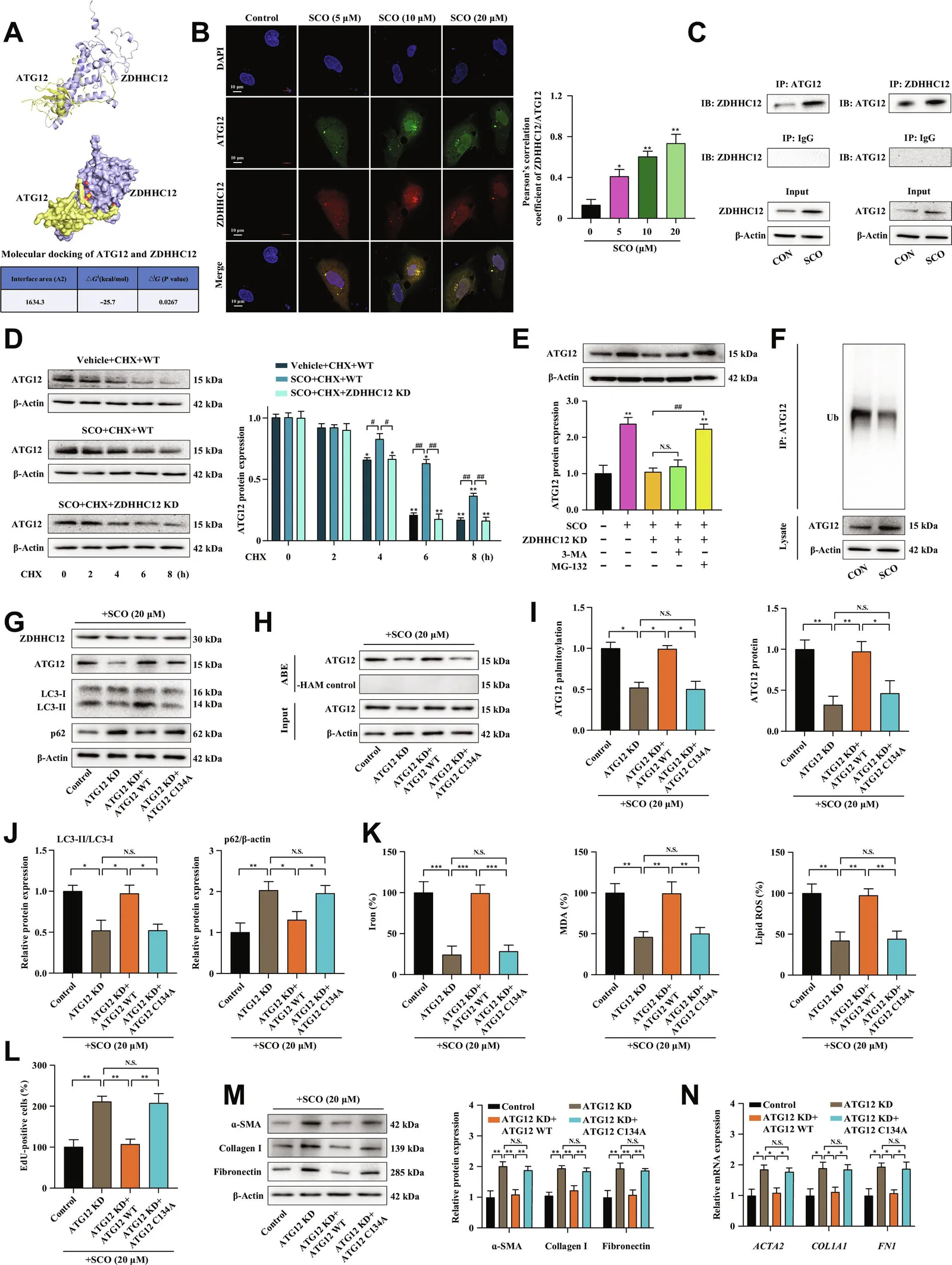
Zinc finger DHHC-type palmitoyltransferase 12 (ZDHHC12) interacts with autophagy-related protein 12 (ATG12) and promotes its S-palmitoylation at Cys134 to modulate ferroptosis in scoparone (SCO)-treated hepatic stellate cells (HSCs). (A) Molecular docking was used to predict the interaction between ATG12 and ZDHHC12. HSC-LX2 cells were exposed to SCO (5, 10, and 20 μM) for 24 h. (B) Subcellular localization of ATG12 and ZDHHC12 was examined by confocal microscopy. Scale bar: 10 μm (*n* =3, ^*^P < 0.05, ^**^P < 0.01). (C) The interaction between ATG12 and ZDHHC12 was determined by coimmunoprecipitation (*n* = 3). (D) HSC-LX2 cells stably transfected with ZDHHC12 short hairpin RNA (shRNA) (knockdown [KD]) were exposed to SCO (20 μM) and cycloheximide (CHX, 50 μg/ml) for 0, 2, 4, 6, and 8 h. CHX chase assay was used to assess ATG12 protein stability (*n* = 3, ^*^P < 0.05, ^**^P < 0.01 compared with 0 h within the same group, ^#^*P* < 0.05, ^##^*P* < 0.01 compared between groups at the same time point). (E) HSC-LX2 cells stably transfected with ZDHHC12 KD were exposed to SCO (20 μM) with 3-methyladenine (3-MA) (5 mM) or MG-132 (10 μM) for 24 h. The protein expression of ATG12 was examined by western blotting (*n* = 3, ^**^P < 0.01 compared with the Control group, ^##^*P* < 0.01 compared with the indicated group). (F) ATG12 ubiquitination levels were assessed by immunoprecipitation followed by immunoblotting (*n* = 3). Rescue experiments were performed by reexpressing ATG12 or ATG12 C134A mutant in ATG12 KD cells, followed by treatment with SCO (20 μM) for 24 h. (G and J) The expression of ZDHHC12, ATG12, LC3-I/II, and p62 was determined by western blotting (*n* = 3, ^*^P < 0.05, ^**^P < 0.01, N.S., not significant). (H and I) The ATG12 S-palmitoylation was examined by the acyl-biotin exchange assay (*n* = 3, ^*^P < 0.05, ^**^P < 0.01, N.S., not significant). (K) The intracellular iron, lipid reactive oxygen species (ROS), and malondialdehyde (MDA) levels were detected by commercial assay kits (*n* = 3, ^*^P < 0.05, ^**^P < 0.01, ^***^P < 0.001, N.S., not significant). (L) Cell proliferation was assessed by the 5-ethynyl-2′-deoxyuridine (EdU) assay (*n* = 3, ^*^P < 0.05, ^**^P < 0.01, N.S., not significant). (M and N) The expression of α-smooth muscle actin (α-SMA), collagen I, and fibronectin was determined by western blot and quantitative real-time polymerase chain reaction (RT-qPCR) (*n* = 3, ^*^P < 0.05, ^**^P < 0.01, N.S., not significant).

### Phase separation of ZDHHC12 enhances ATG12 S-palmitoylation and autophagy activation in SCO-induced HSC ferroptosis

It is widely accepted that LLPS enhances enzymatic catalysis by concentrating reactants into dense condensates, thereby increasing the effective local concentrations of enzymes and substrates and promoting their interaction frequency [33]. We therefore examined whether SCO induces LLPS of ZDHHC12 and enhances its catalytic capacity for S-palmitoylation. Using the FuzDrop algorithm, we first evaluated the intrinsic phase-separation propensity of ZDHHC12. Residue-based droplet-promoting scores predicted residues 84 to 90 as a putative droplet-promoting region (Fig. 6A). Importantly, the amino acid sequence of the ZDHHC12 region spanning residues 84 to 90 was highly conserved across multiple species (Fig. 6B). Confocal imaging revealed that SCO exposure induced the formation of distinct enhanced green fluorescent protein (EGFP)–ZDHHC12 puncta in primary hHSCs and HSC-LX2 cells (Fig. 6C and Fig. S1I). Time-lapse confocal imaging further showed that EGFP–ZDHHC12 puncta underwent dynamic fusion events within seconds, supporting the liquid-like nature of SCO-induced ZDHHC12 condensates (Fig. 6D). Moreover, fluorescence recovery after photobleaching (FRAP) analysis demonstrated that bleached EGFP–ZDHHC12 condensates rapidly recovered fluorescence and gradually reached a substantial recovery level, indicating the dynamic, reversible, and liquid-like properties characteristic of LLPS (Fig. 6E). Treatment with 1,6-hexanediol (1,6-HD) reduced the number and intensity of EGFP–ZDHHC12 puncta compared with the control, suggesting that the condensates were sensitive to disruption of weak hydrophobic interactions (Fig. 6F). To directly evaluate the phase-separation behavior of ZDHHC12, we performed cell-free LLPS assays using purified EGFP–ZDHHC12 protein. The in vitro LLPS assay showed that EGFP–ZDHHC12 formed condensates, and the number of droplets increased as the protein concentration rose from 10 to 80 μM (Fig. 6G). An increase in droplet number was observed as the NaCl concentration decreased from 500 to 37.5 mM, suggesting that lower ionic strength favors condensate formation (Fig. 6H). Moreover, droplet formation was enhanced under acidic pH and physiological temperature, indicating that the phase behavior of ZDHHC12 is sensitive to environmental conditions (Fig. S7A and B). Increasing molecular crowding using polyethylene glycol 8000 (PEG8000) enhanced droplet formation, supporting the intrinsic phase-separation propensity of ZDHHC12 (Fig. S7C). To biochemically validate whether ZDHHC12 condensates enhance its palmitoyltransferase activity, we performed cell-free palmitoyltransferase assays using purified recombinant proteins. Radiolabeled [^3^H]-palmitoyl-CoA incorporation assays demonstrated increased palmitate transfer to ATG12 when ZDHHC12 formed condensates, whereas deletion of residues 84 to 90 (ZDHHC12Δ84–90) reduced radiolabel incorporation (Fig. S7D). Click chemistry-based analysis using 17-ODYA-CoA revealed elevated incorporation of palmitate into ATG12 under condensate-forming conditions, but ZDHHC12Δ84–90 weakened the biotinylation signal (Fig. S7E). Interestingly, western blot analysis indicated that ZDHHC12 protein expression was increased following SCO treatment. ZDHHC12-WT or ZDHHC12Δ84–90 resulted in comparable protein expression levels (Fig. 6I and Fig. S7F). ABE assays showed that SCO treatment increased ATG12 S-palmitoylation compared with the control group. ZDHHC12-WT enhanced SCO-induced ATG12 S-palmitoylation, whereas ZDHHC12Δ84–90 failed to enhance the effect (Fig. 6J and Fig. S7G). Consistently, ATG12 protein expression was increased following SCO treatment. Ectopic expression of ZDHHC12-WT further elevated ATG12 protein levels, while ZDHHC12Δ84–90 did not produce an additional increase in ATG12 expression (Fig. 6I and Fig. S7H). Furthermore, SCO treatment increased the LC3-II/LC3-I ratio and decreased p62 accumulation. When ZDHHC12-WT was overexpressed, the LC3-II/LC3-I ratio was further increased and p62 levels were further reduced under SCO treatment, whereas ZDHHC12Δ84–90 did not augment these autophagy-related changes (Fig. 6I and Fig. S7H). In addition, SCO treatment increased iron accumulation, MDA levels, and lipid ROS. ZDHHC12-WT further potentiated these ferroptosis-associated changes, whereas ZDHHC12Δ84–90 failed to promote ferroptosis-related alterations (Fig. 6K). SCO treatment reduced the protein and mRNA expression of HSC activation markers, including α-SMA, collagen I, and fibronectin. Ectopic expression of ZDHHC12-WT further suppressed the expression of these fibrogenic markers, whereas ZDHHC12Δ84–90 did not confer additional inhibitory effects (Fig. 6L). Collectively, these data suggest that SCO promotes ZDHHC12 LLPS, facilitating ATG12 S-palmitoylation and autophagy-associated ferroptotic responses.

**Fig. 6.**
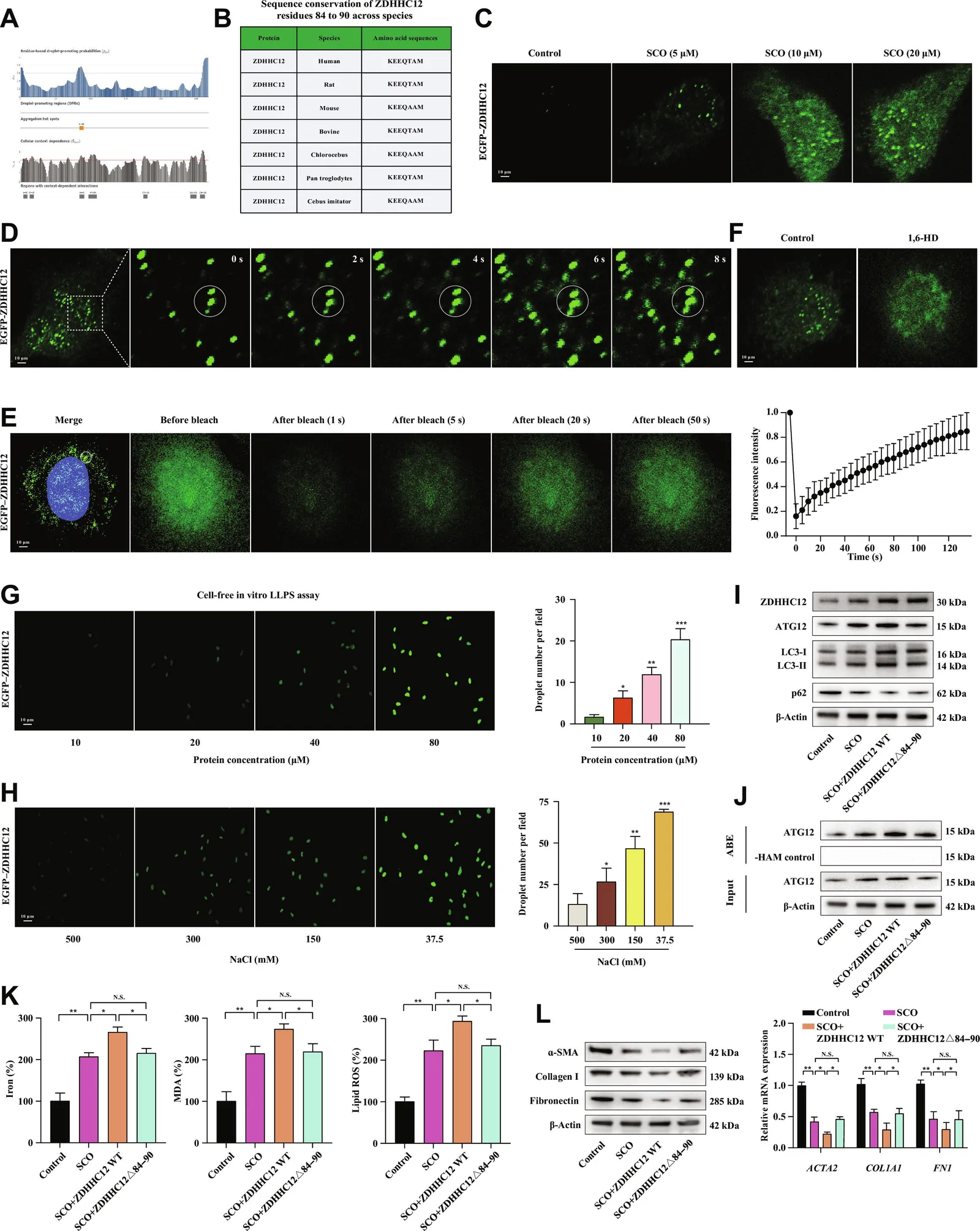
The phase separation of zinc finger DHHC-type palmitoyltransferase 12 (ZDHHC12) regulates palmitoylation-dependent ferroptosis in scoparone (SCO)-treated hepatic stellate cells (HSCs). (A) Computational prediction of intrinsically droplet-promoting regions and aggregation hot spots within ZDHHC12. (B) Sequence alignment of residues 84 to 90 of ZDHHC12 across multiple species. (C) HSC-LX2 cells were exposed to SCO (5, 10, and 20 μM) for 24 h. Fluorescence microscopy images of enhanced green fluorescent protein (EGFP)-tagged ZDHHC12 were examined. Scale bar: 10 μm (*n* = 3). (D) Time-lapse imaging of EGFP–ZDHHC12 condensates was used to demonstrate dynamic droplet behavior. Scale bar: 10 μm (*n* = 3). (E) Fluorescence recovery after photobleaching (FRAP) analysis was used to assess the mobility of ZDHHC12 condensates. Scale bar: 10 μm (*n* = 3). (F) Treatment with 1,6-hexanediol (1,6-HD) disrupted weak hydrophobic interactions to show dissolution of ZDHHC12 puncta. Scale bar: 10 μm (*n* = 3). (G) Purified EGFP–ZDHHC12 protein at the indicated concentrations (10, 20, 40, and 80 μM) was subjected to phase separation under cell-free conditions (*n* = 3, ^*^P < 0.05, ^**^P < 0.01, ^***^P < 0.001 compared with the 10 μM group). (H) Purified EGFP–ZDHHC12 protein was subjected to cell-free liquid–liquid phase separation (LLPS) assays in the presence of different NaCl concentrations (500, 300, 150, and 37.5 mM) (*n* = 3, ^*^P < 0.05, ^**^P < 0.01, ^***^P < 0.001 compared with the 500 mM group). (I) HSC-LX2 cells stably expressing the ZDHHC12 mutant lacking residues 84 to 90 (ZDHHC12Δ84–90) were exposed to SCO (20 μM) for 24 h. The expression of ZDHHC12, autophagy-related protein 12 (ATG12), LC3-I/II, and p62 was determined by western blotting (*n* = 3). (J) The ATG12 S-palmitoylation was examined by the acyl-biotin exchange assay (*n* = 3). (K) The intracellular iron, lipid reactive oxygen species (ROS), and malondialdehyde (MDA) levels were detected by commercial assay kits (*n* = 3, ^*^P < 0.05, ^**^P < 0.01, N.S., not significant). (L) The expression of α-smooth muscle actin (α-SMA), collagen I, and fibronectin was determined by western blot and quantitative real-time polymerase chain reaction (RT-qPCR) (*n* = 3, ^*^P < 0.05, ^**^P < 0.01, N.S., not significant).

### SCO alleviates mouse liver fibrotic injury by promoting HSC ferroptosis through ZDHHC12-dependent ATG12 S-palmitoylation

To validate the antifibrotic role of SCO in vivo, we established 2 independent murine models of liver fibrosis, including carbon tetrachloride (CCl_4_)-induced toxic injury and bile duct ligation (BDL)-induced cholestatic fibrosis [34,35]. Using HSC-targeted liposomal delivery of shRNA (VA-Lip-ZDHHC12 KD and VA-Lip-ATG12 KD), we further investigated whether the therapeutic effect of SCO involves ZDHHC12-mediated ATG12 S-palmitoylation (Fig. S8A and B). Macroscopic examination revealed that SCO treatment improved liver morphology, whereas HSC-specific knockdown of ZDHHC12 or ATG12 diminished this protective effect (Fig. 7A and Fig. S8C). Hematoxylin and eosin (H&E) staining demonstrated that SCO reduced liver architectural damage and inflammatory infiltration, while VA-Lip-ZDHHC12 KD or VA-Lip-ATG12 KD attenuated this improvement (Fig. 7B and Fig. S8D). Consistently, Sirius Red and Masson staining indicated that SCO treatment diminished collagen deposition, but silencing ZDHHC12 or ATG12 reversed the reduction in collagen deposition (Fig. 7B and Fig. S8D). Western blot analysis and immunohistochemical staining showed that SCO treatment decreased the expression of fibrosis-related proteins, including α-SMA, Collagen I and TIMP1, whereas HSC-specific knockdown of ZDHHC12 or ATG12 reversed the inhibitory effect (Fig. 7B and Figs. S8D and S1A). Biochemical analysis showed that SCO administration dynamically decreased serum alanine aminotransferase (ALT), aspartate aminotransferase (AST), and TGF-β1 levels, while VA-Lip-ZDHHC12 KD or VA-Lip-ATG12 KD weakened this hepatoprotective effect (Fig. 7C and Figs. S8E and S9B). We measured the levels of ZDHHC12 and ATG12 in fibrotic and healthy livers. ZDHHC12 and ATG12 were down-regulated in fibrotic liver tissues at both the mRNA and protein levels compared with healthy controls (Fig. S9C). To clarify cell specificity beyond tissue-level histological and biochemical readouts, primary HSCs, hepatocytes, and macrophages were isolated for quantitative analyses. ABE assays demonstrated that SCO treatment increased ATG12 S-palmitoylation in primary HSCs, whereas no obvious changes were observed in hepatocytes or macrophages (Fig. 7D and Figs. S8F and S9D). Western blot analysis further revealed that SCO elevated ZDHHC12 and ATG12 protein levels in primary HSCs but not in hepatocytes or macrophages. VA-Lip-ZDHHC12 KD or VA-Lip-ATG12 KD reduced the corresponding protein levels in primary HSCs, without altering their expression in hepatocytes or macrophages (Fig. 7E and Figs. S8G and S9E). Moreover, SCO treatment increased the LC3-II/LC3-I ratio and reduced p62 protein levels, whereas HSC-specific knockdown of ZDHHC12 or ATG12 attenuated these effects in primary HSCs but not in hepatocytes or macrophages (Fig. 7E and Figs. S8G and S9F). In addition, SCO treatment enhanced ferroptotic events in primary HSCs rather than in hepatocytes or macrophages, as evidenced by increased 4-hydroxynonenal staining, elevated iron deposition, up-regulated PTGS2 expression, reduced GPX4 activity, increased MDA levels, and GSH depletion. These effects were abolished by VA-Lip-ZDHHC12 KD or VA-Lip-ATG12 KD (Fig. 7F to H and Fig. S8H and I). Overall, these results suggest that SCO attenuates hepatic fibrotic injury in mice by inducing ferroptosis in HSCs via ZDHHC12-mediated ATG12 S-palmitoylation.

**Fig. 7.**
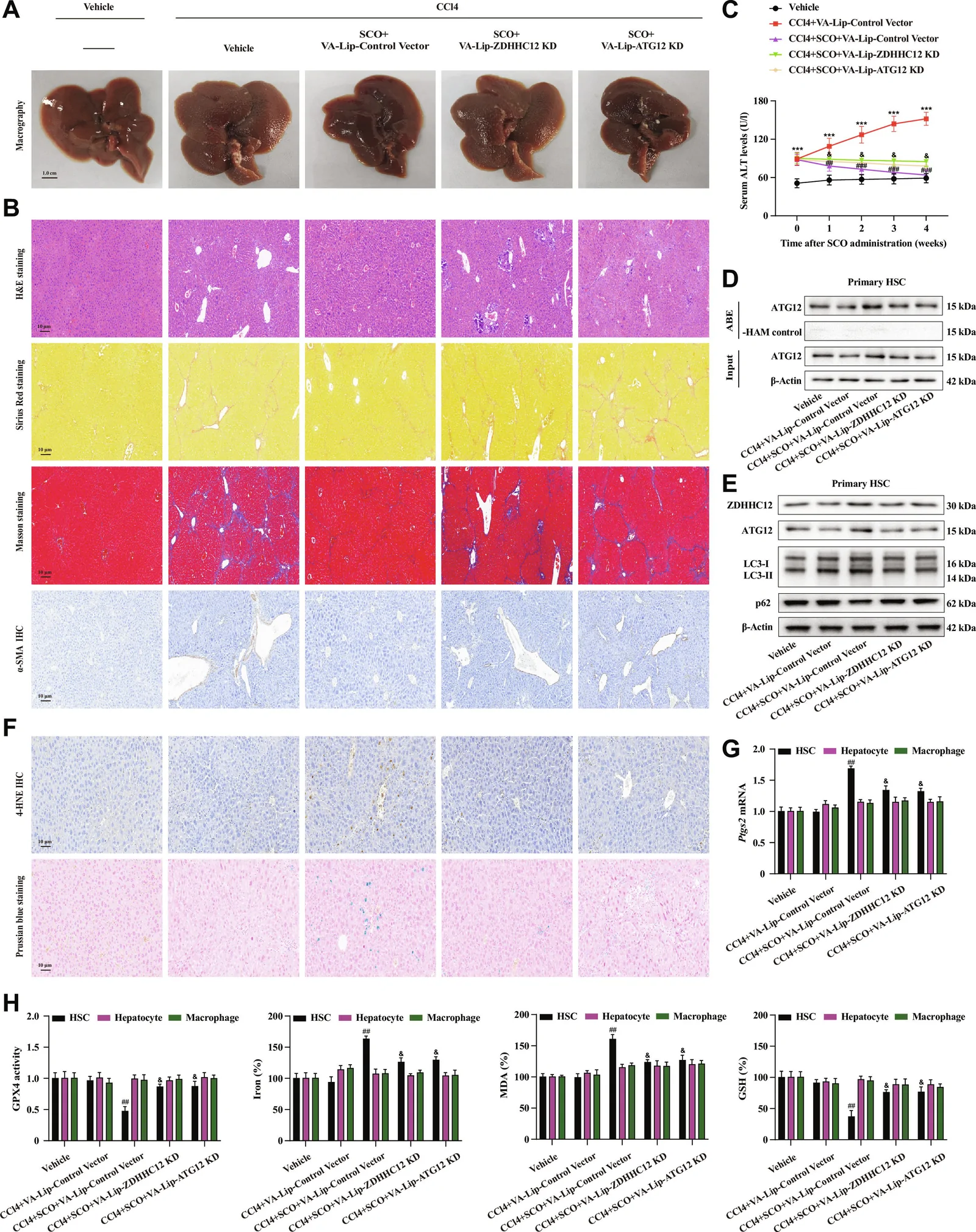
Scoparone (SCO) alleviates liver fibrosis by inducing hepatic stellate cell (HSC) ferroptosis through zinc finger DHHC-type palmitoyltransferase 12 (ZDHHC12)-mediated autophagy-related protein 12 (ATG12) S-palmitoylation. A carbon tetrachloride (CCl_4_)-induced liver fibrosis mouse model was established, followed by administration of Vehicle, CCl_4_+VA-Lip-Control Vector, CCl_4_+SCO+VA-Lip-Control Vector, CCl_4_+SCO+VA-Lip-ZDHHC12 knockdown (KD), or CCl_4_+SCO+VA-Lip-ATG12 KD. (A) Representative gross morphology of liver tissues. Scale bar: 1 cm (*n* = 8). (B) Hematoxylin and eosin (H&E), Sirius Red, Masson, and α-smooth muscle actin (α-SMA) immunohistochemical staining were performed. Scale bar: 10 μm (*n* = 8). (C) Serum alanine aminotransferase (ALT) levels were detected by serum biochemical analysis (*n* = 8, statistical comparisons were performed among groups at the same time point. ^***^P < 0.001 compared with the Vehicle group, ^##^*P* < 0.01, ^###^*P* < 0.001 compared with the CCl_4_+VA-Lip-Control Vector group, ^&^*P* < 0.05 compared with the CCl_4_+SCO+VA-Lip-Control Vector group). (D) ATG12 S-palmitoylation levels in primary HSCs were determined by acyl-biotin exchange assay (*n* = 8). (E) The expression of ZDHHC12, ATG12, LC3-I/II, and p62 in primary HSCs was determined by western blotting (*n* = 8). (F) Immunohistochemical staining for 4-hydroxynonenal and Prussian blue staining were used to evaluate lipid peroxidation and iron deposition. Scale bar: 10 μm (*n* = 8). (G) The expression of *Ptgs2* was determined by quantitative real-time polymerase chain reaction (RT-qPCR) (*n* = 8, ^##^*P* < 0.01 compared with the CCl_4_+VA-Lip-Control Vector group, ^&^*P* < 0.05 compared with the CCl_4_+SCO+VA-Lip-Control Vector group). (H) Glutathione peroxidase 4 (GPX4) activity was measured using a GPX4 activity assay kit. The intracellular iron, malondialdehyde (MDA), and glutathione (GSH) levels were examined by commercial assay kits (*n* = 8, ^##^*P* < 0.01 compared with the CCl_4_+VA-Lip-Control Vector group, ^&^*P* < 0.05 compared with the CCl_4_+SCO+VA-Lip-Control Vector group).

### ZDHHC12 expression positively correlates with ATG12 expression and is associated with fibrosis progression in patients with liver fibrosis

To explore the clinical relevance of the ZDHHC12–ATG12 axis in human liver fibrosis, we first interrogated publicly available transcriptomic datasets at single-cell resolution. Uniform manifold approximation and projection visualization delineated distinct hepatic cell clusters, including hepatocytes, HSCs, macrophages, endothelial cells, and various immune populations (Fig. 8A). Feature plot analysis further demonstrated that ZDHHC12 and ATG12 exhibited altered expression patterns in fibrotic liver tissues compared with healthy controls, with partially overlapping distributions localized to nonparenchymal cell populations (Fig. 8B and C). Moreover, ZDHHC12 and ATG12 expression decreased with advancing fibrosis stage from F1 to F4 (Fig. 8D). Correlation analysis demonstrated a positive correlation between ZDHHC12 and ATG12 expression in liver fibrosis samples (Fig. 8E). Pseudotime trajectory analysis showed that ZDHHC12 and ATG12 expression scores changed along fibrosis progression, with temporal variation in their respective clusters, suggesting coordinated regulation during disease development (Fig. 8F). Furthermore, gene set enrichment analysis indicated that hepatic fibrosis-related gene sets were significantly enriched in the ZDHHC12-low and ATG12-low subgroups, whereas ferroptosis-related gene sets were enriched in the ZDHHC12-high and ATG12-high subgroups (Fig. 8G and H). Kaplan–Meier survival analysis suggested that lower ZDHHC12 or ATG12 expression was associated with less favorable survival outcomes (Fig. 8I and J). Notably, the association between low ZDHHC12 expression and reduced cirrhosis-free survival was statistically borderline by the 2-sided Mantel–Cox log-rank test (*P* = 0.049; hazard ratio = 1.4). To further examine the borderline association between low ZDHHC12 expression and cirrhosis-free survival, we performed exploratory complementary tests using alternative event-weighting schemes. These analyses yielded directionally consistent results, including the Gehan–Breslow–Wilcoxon test (*P* = 0.036) and the Tarone–Ware test (*P* = 0.041). In addition, receiver operating characteristic curve analysis revealed that ZDHHC12 and ATG12 exhibited diagnostic performance (Fig. 8K). The multivariable Cox proportional hazards model adjusted for key clinicopathologic factors showed that ZDHHC12 and ATG12 were independent prognostic factors (Fig. 8L). To further strengthen the clinical validity beyond public transcriptomic datasets, we analyzed an independent cohort comprising 40 patients with histologically confirmed liver fibrosis and nonfibrotic liver tissues. Both ZDHHC12 and ATG12 were reduced in fibrotic liver tissues compared with nonfibrotic liver tissues (Fig. S10A). Correlation analysis within fibrotic specimens confirmed a positive relationship between ZDHHC12 and ATG12 expression levels (Fig. S10B). Moreover, stratification by METAVIR stage revealed a progressive decrease in ZDHHC12 and ATG12 from F1 to F4. Linear trend analysis further indicated a significant decreasing trend of both ZDHHC12 and ATG12 expression with increasing fibrosis stage (Fig. S10C). Linear regression analyses demonstrated that ZDHHC12 and ATG12 expression levels were inversely correlated with serum ALT and AST concentrations (Fig. S10D). Multivariate logistic regression analysis showed that decreased ZDHHC12 and ATG12 expression was independently associated with advanced fibrosis (Fig. S10E). Altogether, these findings indicate that reduced expression of ZDHHC12 and ATG12 is associated with fibrosis progression and adverse prognosis in liver fibrosis.

**Fig. 8.**
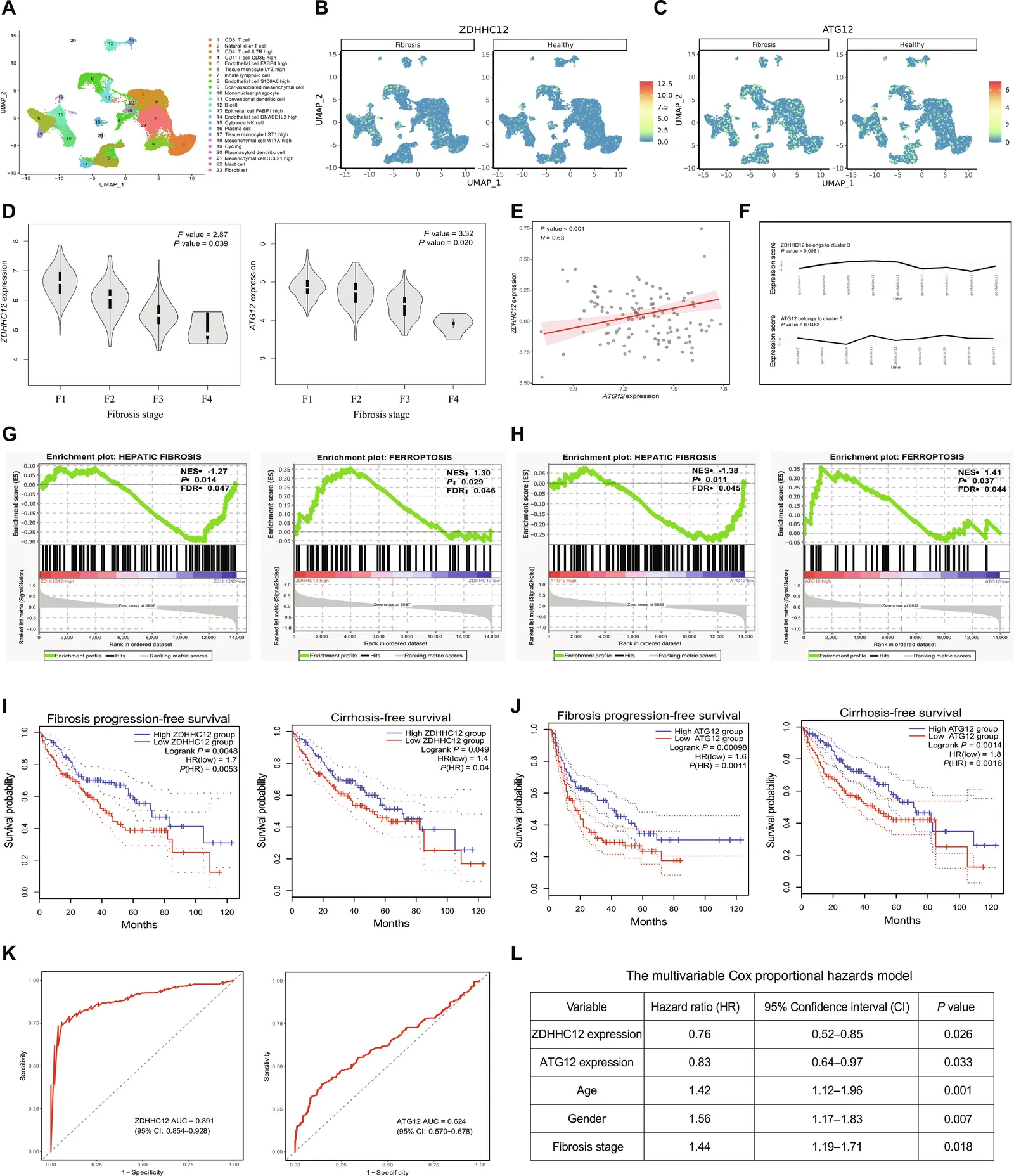
Clinical relevance of zinc finger DHHC-type palmitoyltransferase 12 (ZDHHC12) and autophagy-related protein 12 (ATG12) expression in liver disease datasets. (A) Uniform manifold approximation and projection (UMAP) visualization showing distinct hepatic cell populations. (B and C) Feature plots illustrating ZDHHC12 and ATG12 expression. (D) Violin plots showing ZDHHC12 and ATG12 expression across fibrosis stages. Stages F0 (*n* = 43), F1 (*n* = 20), F2 (*n* = 33), F3 (*n* = 18), and F4 (*n* = 10). (E) Correlation analysis between ZDHHC12 and ATG12 expression (*n* = 124). (F) Pseudotime analysis showing dynamic expression patterns. (G and H) Gene set enrichment analysis (GSEA) was used to show significant enrichment of hepatic fibrosis and ferroptosis pathways in samples with ZDHHC12 or ATG12 expression. (I and J) Kaplan–Meier survival curves for fibrosis progression-free survival and cirrhosis-free survival were stratified by ZDHHC12 or ATG12 expression. (K) Receiver operating characteristic (ROC) curve analysis was used to evaluate the diagnostic performance of ZDHHC12 and ATG12. (L) Multivariate Cox proportional hazards regression analysis was used to identify ZDHHC12 and ATG12 expression as independent prognostic factors. NES, normalized enrichment score; FDR, false discovery rate; AUC, area under the curve.

## Discussion

Activated HSCs are central drivers of extracellular matrix accumulation during chronic liver injury, and their selective elimination has emerged as a potential strategy for fibrosis regression [36]. Among these processes, ferroptosis has attracted increasing interest because activated HSCs exhibit heightened sensitivity to perturbations in iron handling, lipid peroxidation dynamics, and intracellular redox buffering [37]. Previous studies have reported that pharmacological agents such as sorafenib and celastrol can reduce experimental fibrosis in association with enhanced ferroptotic susceptibility of HSCs [38,39]. These studies have supported a functional connection between HSC ferroptosis and fibrosis resolution. However, most have concentrated on relatively downstream components of the ferroptotic machinery, including GPX4, SLC7A11, ACSL4, FTH1, and Beclin 1 (BECN1) [37–39]. How upstream metabolic changes are transmitted to the core autophagy machinery to influence ferroptosis remains less well understood. The present study extends this framework by suggesting a mechanism that connects SCO-induced lipid metabolic remodeling with the posttranslational regulation of ATG12. Specifically, SCO treatment increased the cellular C16 fatty acid pool and ZDHHC12 expression and promoted the assembly of ZDHHC12 into dynamic condensates. This organization appeared to facilitate ATG12 S-palmitoylation at Cys134, limit its ubiquitin–proteasome-dependent degradation, and support ATG12–ATG5 conjugate formation and LC3 lipidation. These changes were accompanied by enhanced FTH1 degradation, iron release, lipid peroxidation, and ferroptotic features in activated HSCs. Silencing ZDHHC12, deleting its LLPS-associated 84–90 region, or mutating ATG12 Cys134 attenuated several of these responses. Its biological relevance was further supported by HSC-targeted knockdown experiments in fibrotic mice and by replication of key observations in primary hHSCs. Compared with earlier studies centered on downstream ferroptosis effectors, our results suggest that condensate-associated S-palmitoylation of an autophagy protein may represent an additional regulatory layer linking metabolic cues to ferroptosis.

S-palmitoylation has attracted increasing attention in ferroptosis regulation because this lipid modification can dynamically alter the stability, localization, and function of proteins that govern cellular redox balance and lipid peroxide metabolism [40,41]. Previous investigations have primarily linked S-palmitoylation to proteins that directly govern antioxidant capacity or lipid peroxide production. S-palmitoylation-dependent stabilization of GPX4 or SLC7A11 has been reported to reinforce antioxidant defense and reduce ferroptotic sensitivity, whereas regulation of ubiquitin-specific peptidase 19 through S-palmitoylation may limit ACSL4-driven lipid peroxidation in cancer cells [42,43]. Although these studies have broadened our understanding of S-palmitoylation in established ferroptosis-related pathways, it remains unclear whether this modification targets core autophagy proteins to regulate ferritin turnover and intracellular iron availability. In this study, ZDHHC12 was identified as a palmitoyltransferase involved in the ferroptotic response of activated HSCs. ZDHHC12 knockdown attenuated SCO-induced iron accumulation and lipid peroxidation, whereas ZDHHC12 OE enhanced these responses. We further identified ATG12 as a candidate substrate of ZDHHC12, extending the potential scope of ferroptosis-related S-palmitoylation beyond antioxidant and lipid-redox proteins to the autophagy machinery. Site-prediction and mutational analyses indicated that the conserved Cys134 residue is important for ATG12 S-palmitoylation. Limited proteolysis and thermal stability analyses suggested that modification at Cys134 may stabilize a conformational state of ATG12 that favors its assembly with ATG5. Moreover, S-palmitoylation has been reported to regulate ubiquitination-dependent degradation of substrate proteins by altering protein conformation, local hydrophobicity, or steric accessibility, thereby influencing the accessibility of substrates to ubiquitination machinery. In line with these observations, SCO-induced ATG12 S-palmitoylation was associated with reduced ATG12 ubiquitination and increased ATG12 stability. Furthermore, the ATG12-Cys134A mutant displayed higher ubiquitination levels than ATG12-WT following SCO treatment, supporting the possibility that Cys134 palmitoylation reduces the susceptibility of ATG12 to ubiquitination-dependent turnover. This mechanism provides a possible link between protein lipidation and autophagy-dependent ferritin turnover, thereby offering an additional explanation for how intracellular iron availability is regulated during ferroptosis.

LLPS can organize biochemical reactions by concentrating selected molecules within dynamic compartments, thereby changing their local availability and interaction kinetics [44,45]. Previous studies linking LLPS with S-palmitoylation have mainly examined how lipid modification affects the localization or condensate behavior of substrate proteins. The S-palmitoylation cycle of p62 regulates its recruitment to autophagic membranes, whereas ZDHHC7-mediated S-palmitoylation drives NLRP3 phase separation and inflammasome activation [46,47]. In ferroptosis, ferroptosis suppressor protein 1 phase separation can alter its membrane distribution and compromise its lipid peroxide-reducing activity [48]. However, whether phase separation of a palmitoyltransferase itself can serve as an upstream catalytic platform for ferroptosis regulation has remained unclear. Our study addresses this gap by demonstrating that SCO can promote dynamic ZDHHC12 puncta displaying fusion and fluorescence recovery, while purified recombinant ZDHHC12 formed condensates in vitro in a manner sensitive to protein concentration, ionic strength, pH, temperature, and molecular crowding. Deletion of the conserved 84–90 region reduced condensate formation and ATG12 S-palmitoylation, with corresponding attenuation of autophagic and ferroptotic responses in HSCs. More importantly, given that ZDHHC12 is a palmitoyl acyltransferase and ZDHHC family enzymes undergo autopalmitoylation at conserved cysteine residues within their DHHC motif [49], it is essential to investigate whether ZDHHC12 autopalmitoylation influences its phase separation behavior and consequently regulates downstream palmitoylation of ATG12. In future research, we will explore ZDHHC12 autopalmitoylation under SCO-treated conditions through ABE and metabolic labeling methods. We will then generate catalytic cysteine mutants within the DHHC motif to abolish autopalmitoylation and evaluate whether loss of the thioester intermediate impairs condensate formation, droplet dynamics, and FRAP recovery. In addition, the conserved DHHC catalytic cysteine is located within the cytosolic catalytic loop, whereas the LLPS-promoting 84–90 region is spatially distinct based on domain organization. We will evaluate whether the Δ84–90 mutant affects autopalmitoylation levels to avoid confounding effects between altered LLPS properties and changes in catalytic activity.

SCO is a naturally occurring coumarin characterized by a benzopyrone core bearing adjacent methoxy groups at the C6 and C7 positions. This relatively simple scaffold distinguishes SCO from more structurally complex natural products and may contribute to its broad biological activity [20]. SCO was selected for this study primarily because it is a major bioactive constituent of *Artemisia scoparia*, which has traditionally been used for hepatobiliary disorders [21,22]. Moreover, our previous work showed that SCO can attenuate HSC activation and experimental liver fibrosis [23]. Earlier studies have generally attributed its hepatoprotective effects to the modulation of inflammation, oxidative stress, bile acid homeostasis, and metabolic signaling [50,51]. In the present study, we reported that SCO suppressed HSC proliferation and fibrogenic activation by inducing ferroptosis in vitro, whereas pharmacological inhibition of ferroptosis partially reversed these effects. Consistently, SCO alleviated liver injury, collagen deposition, and HSC activation in experimental models of liver fibrosis. Analyses of isolated hepatic cell populations further indicated that SCO-induced ferroptotic responses occurred predominantly in HSCs, with comparatively limited changes in hepatocytes and macrophages. Mechanistically, SCO enhanced de novo palmitate synthesis potentially through the mTOR–SREBP1–ACC signaling pathway, thereby expanding the cellular C16 fatty acid pool available for protein S-palmitoylation. This metabolic change was accompanied by increased ZDHHC12 expression, ZDHHC12 condensate formation, and ATG12 S-palmitoylation, ultimately favoring ferritinophagy-associated iron mobilization. The attenuation of SCO-induced ferroptosis and fibrosis improvement following HSC-targeted silencing of ZDHHC12 or ATG12 supports the contribution of this pathway to its pharmacological effects. With further mechanistic and preclinical validation, SCO may represent a promising candidate for the development of antifibrotic therapies.

Several limitations should be considered when interpreting these findings. First, although SCO showed antifibrotic activity in experimental mouse models, its efficacy and safety have not yet been evaluated in patients with liver fibrosis. Differences in drug exposure, disease etiology, fibrosis stage, and hepatic metabolism may limit the direct translation of findings from animal models to humans. Future studies should therefore define the pharmacokinetic profile, long-term safety, optimal dose, and therapeutic window of SCO before clinical evaluation. Second, the clinical validation was based on a relatively small cohort of 40 patients with liver fibrosis. Although the observed changes in ZDHHC12 and ATG12 were consistent with the transcriptomic analyses, the limited sample size and heterogeneous etiologies restrict the generalizability of these findings. Future studies will therefore require larger, well-characterized patient cohorts as well as multicenter and longitudinal validation. Third, the possible contribution of ZDHHC12 autopalmitoylation was not examined in the present study. As a member of the ZDHHC palmitoyltransferase family, ZDHHC12 may undergo autopalmitoylation at the catalytic cysteine within its DHHC motif, generating an acyl-enzyme intermediate required for substrate modification. Whether this process affects ZDHHC12 condensate formation, droplet dynamics, or substrate recognition remains unclear. Future studies using metabolic labeling, ABE assays, and catalytic cysteine mutants will be needed to determine whether ZDHHC12 autopalmitoylation contributes to its phase separation behavior and downstream ATG12 S-palmitoylation. Fourth, although our data suggest that SCO enhances de novo palmitate synthesis potentially through the mTOR–SREBP1–ACC axis, the upstream metabolic regulation remains incompletely defined. The current evidence links SCO treatment with activation of this lipogenic pathway and expansion of the C16 fatty acid pool but does not fully establish whether mTOR–SREBP1–ACC signaling is required for ZDHHC12 condensate formation, ATG12 S-palmitoylation, and ferroptosis induction. Additional gain- and loss-of-function experiments targeting mTOR, SREBP1, and ACC will be necessary to clarify the causal contribution of this pathway. Addressing these limitations will be important for assessing the clinical relevance of SCO and the broader applicability of the proposed pathway.

In summary, our findings suggest that SCO promotes the ferroptotic clearance of activated HSCs through an LLPS-associated ZDHHC12–ATG12 signaling axis. ZDHHC12 condensation facilitates ATG12 S-palmitoylation at Cys134, thereby enhancing ATG12 stability and promoting autophagy-dependent iron mobilization. These events increase lipid peroxidation, suppress HSC activation, and ultimately attenuate liver fibrosis (Fig. 9). This study provides a potential mechanistic link among metabolic remodeling, spatial organization of palmitoylation, autophagy, and ferroptosis. Further investigation is needed to define the physiological relevance and therapeutic potential of this pathway in liver fibrosis.

**Fig. 9.**
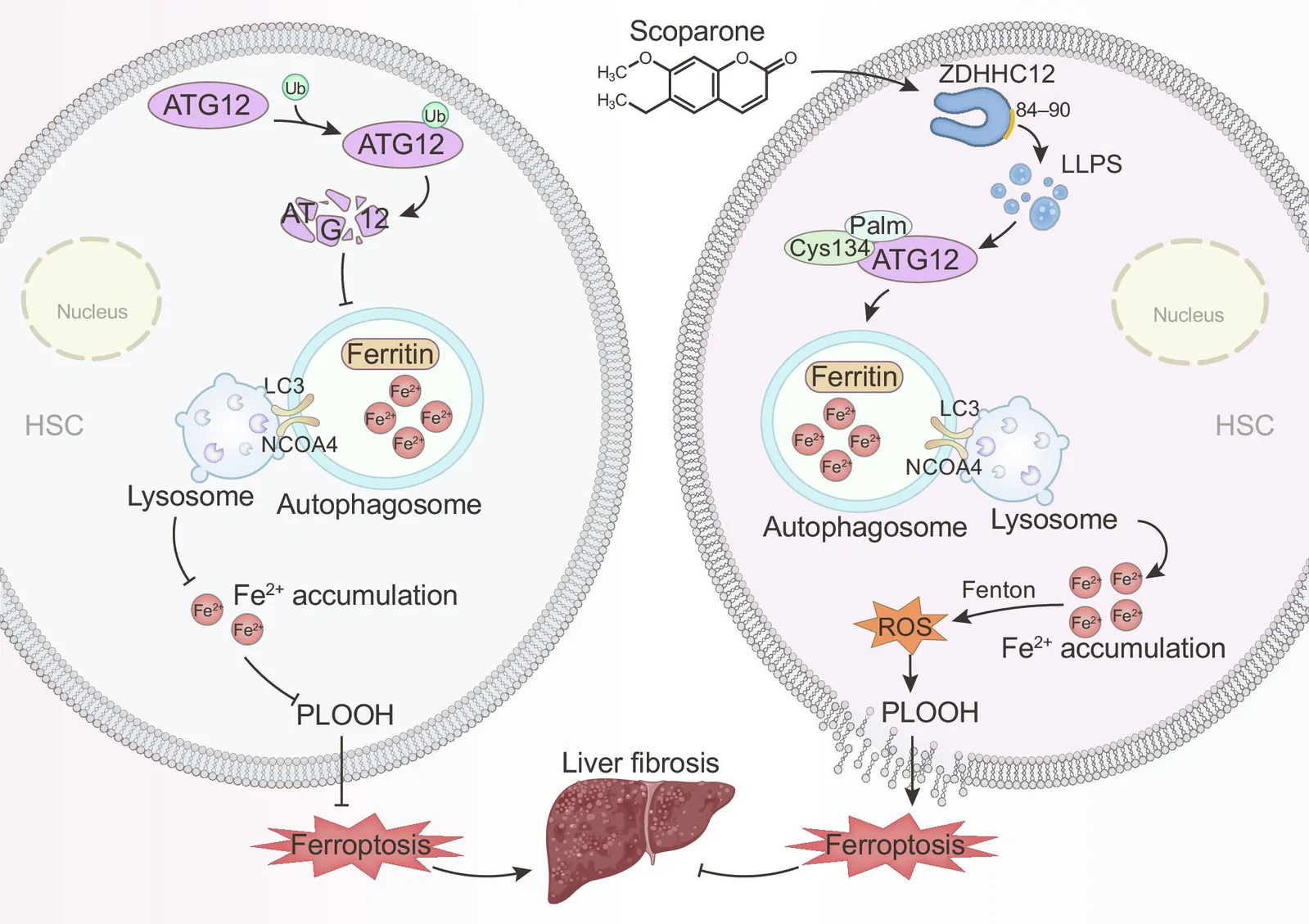
A mechanistic model was used to illustrate the role of the zinc finger DHHC-type palmitoyltransferase 12 (ZDHHC12)–autophagy-related protein 12 (ATG12) axis in scoparone (SCO)-mediated attenuation of liver fibrosis. SCO promotes ZDHHC12 phase separation, thereby facilitating ATG12 S-palmitoylation and autophagy activation, leading to increased ferroptosis sensitivity in hepatic stellate cells (HSCs) and contributing to the attenuation of liver fibrosis.

## Materials and Methods

### Chemicals and antibodies

SCO (S3613), SM (S2358), Fer-1 (S7243), Lip-1 (S7699), 2-BP (E0120), cycloheximide (S7418), MG-132 (S2619), and 3-methyladenine (S2767) were bought from Selleck Chemicals. TGF-β1 (GF346), Fatostatin (341329), Palm B (178501), rapamycin (553210), 1,6-HD (240117), NaCl (S9888), PEG8000 (1546605), and CCl_4_ (79743) were obtained from Sigma-Aldrich. Anti-α-SMA antibody (ab124964), anti-collagen I antibody (ab138492), anti-fibronectin antibody (ab45688), anti-GPX4 antibody (ab125066), anti-SLC7A11 antibody (ab307601), anti-ACSL4 antibody (ab155282), anti-SREBP1 antibody (ab313881), anti-ACC antibody (ab45174), anti-FASN antibody (ab128870), anti-PPARγ antibody (ab45036), anti-p-AMPK antibody (ab131357), anti-AMPK antibody (ab32047), anti-p-mTOR antibody (ab109268), anti-mTOR antibody (ab32028), anti-ZDHHC12 antibody (ab237688), anti-ATG3 antibody (ab108251), anti-ATG4B antibody (ab223364), anti-BECN1 antibody (ab217179), anti-ATG7 antibody (ab133528), anti-ATG9A antibody (ab108338), anti-ATG12–ATG5 antibody (ab303488), anti-ATG14 antibody (ab315009), anti-ATG16L1 antibody (ab187671), anti-LC3I/II antibody (ab62721), anti-p62 antibody (ab109012), anti-FTH1 antibody (ab75973), anti-NLRP3 antibody (ab263899), anti-PARP1 antibody (ab227244), anti-4-hydroxynonenal antibody (ab48506), anti-TIMP1 antibody (ab325798), anti-β-actin antibody (ab8226), anti-mouse immunoglobulin G (IgG) antibody (ab190475), and anti-rabbit IgG antibody (ab288151) were purchased from Abcam Technology. Anti-FAM8A1 antibody (PA5-65387) was obtained from Thermo Fisher Scientific.

### Plasmid construction and stable cell line generation

ZDHHC12 shRNA, ZDHHC12 plasmid, ATG12 plasmid, GFP-LC3 plasmid, RFP-GFP-LC3 plasmid, and control vectors were purchased from Applied Biological Materials. ATG12 Cys134 mutant and ZDHHC12 mutant lacking the 84–90 region (ZDHHC12Δ84–90) were constructed by PCR-based mutagenesis. The primer pairs were as follows: ATG12 Cys134 mutant, forward 5′-CGCAAATGGGCGGTAGGCGTG-3′ and reverse 5′-GAGCCAGTACACGACATCAC-3′; ZDHHC12Δ84–90, forward 5​′-C​AGC​CTC​AGG​AGG​AGC​TCA​AAC​CTC​CAG​CCA​TCCCTCTTCGG-3′ and reverse 5​′-C​CGA​AGA​GGG​ATG​GCT​GGA​GGT​TTG​AGC​TCCTCCTGAGGCT G-3′. VA-Lip-ZDHHC12 KD, VA-Lip-ATG12 KD, and VA-Lip-Control Vector were prepared according to previously established protocols [32]. Briefly, the vitamin A (VA) stock solution was generated by dissolving 5 mg of VA (95144, Sigma) in 50 μl of dimethyl sulfoxide. Subsequently, 0.14 μmol of Lipotrust was mixed with 280 nmol of VA solution in a tube and maintained at 25 °C. Under continuous stirring, 12.24 nmol of the corresponding shRNA, including ZDHHC12 shRNA, ATG12 shRNA, or control shRNA, was added to the VA-Lip mixture to obtain the indicated VA-Lip-shRNA complexes. The resulting preparations were processed through a filter, and the filter-retained material together with the collected fractions was recovered to achieve the required dose for subsequent in vivo administration. All constructs and mutant plasmids were confirmed by DNA sequencing. Stable cell lines were generated by lentiviral transduction followed by puromycin selection at 10 μg/ml, which was initiated 72 h after infection. The efficiencies of knockdown and OE were validated at the mRNA and protein levels using RT-qPCR and western blotting.

### Cell culture

The hHSC-LX2 line (RRID: CVCL_B105) was obtained from the American Type Culture Collection. The cells were plated in Dulbecco’s modified Eagle medium (DMEM) supplemented with 10% fetal bovine serum (FBS) and 1% penicillin–streptomycin at 37 °C with 5% CO_2_.

### EdU assay

Cell proliferation was evaluated using the EdU incorporation assay (Beyotime, C0071S) according to the manufacturer’s instructions. Cells were plated at a density of 5 × 10^4^ in each well of a 24-well plate and allowed to settle overnight. To quantitate DNA synthesis, cells were labeled with EdU (10 μM) in the culture medium for 2 h at 37 °C. After labeling, cells were fixed with 4% paraformaldehyde for 15 min and permeabilized using 0.1% Triton X-100 for 10 min. EdU staining was performed according to the standard protocol, and nuclei were counterstained with 4′,6-diamidino-2-phenylindole. Different fields were taken with a fluorescence microscope using the same exposure time, and EdU-positive cells were calculated according to the number of labeled cells as a percentage of the total nuclei in random fields.

### Cell viability assay

Cell viability was measured using the Cell Counting Kit-8 (Abcam, ab228554). Briefly, 10,000 cells were seeded into 96-well culture plates. After treatment, 10 μl of Cell Counting Kit-8 working solution was added to each well, and the cells were incubated at 37 °C for 4 h. The absorbance was measured at 450 nm using a microplate reader. Cell viability was expressed as a percentage relative to the vehicle-treated control group.

### Iron assay

Cellular iron levels were measured using a commercial Iron Assay Kit (Abcam, ab83366) according to the manufacturer’s instructions. Briefly, cells were lysed in the provided assay buffer and centrifuged at 16,000 × *g* for 10 min at 4 °C to obtain the supernatant. For total iron detection, samples were incubated with iron reducer to convert ferric iron to ferrous iron at 37 °C for 30 min. Subsequently, iron probe was added and samples were incubated for 60 min at room temperature protected from light. Absorbance was measured at 593 nm using a microplate reader. Iron concentrations were calculated based on a standard curve generated using iron standards provided in the kit and normalized to total protein.

### Lipid ROS measurement

Lipid ROS levels were assessed using the ratiometric fluorescent probe C11-BODIPY 581/591 (Thermo Fisher Scientific, D3861). Cells were stained with 2 μM C11-BODIPY for 30 min at 37 °C. The probe can diffuse across the cell membrane and accumulate within the membrane lipids. After staining, the cells were washed 3 times with phosphate-buffered saline (PBS) to remove free C11-BODIPY. The fluorescence was subjected to flow cytometry analysis with 488-nm excitation. The oxidized form of C11-BODIPY was detected in the fluorescein isothiocyanate channel (green fluorescence), and the reduced form was detected in the phycoerythrin channel (red fluorescence). The lipid ROS levels were calculated as the ratio of green fluorescence to red fluorescence intensity.

### Lipid peroxidation assay

Lipid peroxidation was quantified by determining MDA levels using a colorimetric assay kit (Abcam, ab118970). Briefly, cells were harvested and homogenized in lysis buffer on ice, followed by centrifugation at 13,000 × *g* for 10 min at 4 °C to remove insoluble debris. The supernatant was collected and reacted with thiobarbituric acid reagent under high-temperature conditions (95 °C for 60 min) to form the MDA–thiobarbituric acid adduct. After cooling to room temperature, absorbance was measured at 532 nm using a microplate reader. MDA concentrations were calculated from a standard calibration curve generated with MDA standards and normalized to total protein content.

### GSH assay

Intracellular GSH levels were determined using a colorimetric assay kit (Abcam, ab112132). Cells were harvested and resuspended in ice-cold 50 mM MES buffer to maintain thiol stability, followed by centrifugation at 10,000 × *g* for 15 min at 4 °C to remove cellular debris. The supernatants were then used for the GSH reaction mixture. After reacting with the color reagent at room temperature for 25 min, the absorbance at 412 nm was read in a microplate reader. GSH concentrations were determined based on a standard GSH calibration curve and were normalized to total protein.

### GPX4 activity assay

GPX4 activity was measured according to the nicotinamide adenine dinucleotide phosphate hydrogen (NADPH) consumption using a colorimetric assay kit (Sigma-Aldrich, CGP1). Cells were washed twice with ice-cold PBS and lysed using the extraction buffer from the kit. Insoluble material was removed by centrifugation at 12,000× *g* for 10 min at 4 °C. Protein was determined by the bicinchoninic acid (BCA) test, and the amounts used in the assay were as identified per the instructions. The reaction mixture contained GPX assay buffer, NADPH, glutathione, and glutathione reductase as per the manufacturer’s specifications. The reaction was initiated by adding peroxide substrate, and the decrease in NADPH absorbance was read at 340 nm every minute for 5 min on a microplate reader. The enzyme activity was assessed according to the linear rate of oxidation of NADPH as instructed by the manufacturer and expressed as units per milligram of protein.

### Western blot analysis

Total protein was extracted using lysis buffer, and protein concentrations were determined using the BCA assay. Equal amounts of protein were mixed with 5× Laemmli sample buffer and boiled at 95 °C for 15 min. Proteins were separated by SDS-polyacrylamide gel electrophoresis and transferred onto polyvinylidene difluoride membranes. The membranes were blocked with 5% skimmed milk in tris-buffered saline with Tween 20 for 30 min at room temperature and then incubated overnight at 4 °C with the indicated primary antibodies. After washing with tris-buffered saline with Tween 20, the membranes were incubated with horseradish peroxidase-conjugated secondary antibodies for 1 h at 37 °C. Immunoreactive bands were visualized using enhanced chemiluminescence reagents, and densitometric analysis was performed using ImageJ software.

### Immunoprecipitation

Cells were lysed using NP-40 lysis buffer containing a protease inhibitor cocktail on ice, followed by centrifugation. The protein concentration was measured using the BCA assay. Using equal protein amounts, immunoprecipitations were performed with specific primary antibodies at 4 °C overnight by rotating them to allow immune complex formation. Immune complexes were then captured using either Protein A/G agarose beads or magnetic beads for 2 h at 4 °C. Beads with attached immune complexes were washed with lysis buffer to remove nonspecific material. Proteins bound to beads were eluted in SDS sample buffer by boiling, subjected to sodium dodecyl sulfate-polyacrylamide gel electrophoresis (SDS-PAGE), transferred to membranes, and immunoblotted with primary antibodies. Normal IgG was used as the negative control, while inputs were run in parallel to verify the relative expression levels of the proteins.

### ABE assay

Cells were lysed in a buffer supplemented with N-ethylmaleimide to irreversibly block free cysteine thiol groups. After removing excess N-ethylmaleimide by precipitation and washing, thioester-linked palmitoyl groups were selectively cleaved by incubating with hydroxylamine. Then, the newly exposed cysteine residues were labeled with a sulfhydryl-reactive biotin reagent. Biotinylated proteins were pulled down using streptavidin–agarose beads, eluted, and analyzed by SDS-PAGE followed by immunoblotting or mass spectrometry. Parallel samples processed without hydroxylamine served as negative controls to verify the specificity of S-palmitoylation detection.

### Ubiquitination assay

Cells were lysed in denaturing lysis buffer supplemented with protease inhibitors and deubiquitinase inhibitors to preserve ubiquitin modifications. Lysates were clarified by centrifugation, and equal amounts of protein were used for immunoprecipitation. For ubiquitination detection, lysates were incubated overnight at 4 °C with either an anti-ubiquitin antibody or an antibody against the protein of interest. Immune complexes were captured using Protein A/G agarose beads and extensively washed to minimize nonspecific binding. Bound proteins were eluted in SDS sample buffer and subjected to SDS-PAGE followed by immunoblot analysis with the indicated antibodies.

### RT-qPCR

Total RNA was isolated from the cells using the RNA Isolation Kit (Beyotime, R0018M) according to the manufacturer’s instructions. Reverse transcription of purified RNA was performed to synthesize complementary DNA. Real-time PCR amplification was then performed using the SYBR Green detection system on a StepOne Real-Time PCR instrument (Applied Biosystems). Relative mRNA expression was normalized to glyceraldehyde phosphate dehydrogenase, and fold changes were calculated using the 2^−ΔΔCt^ method. Detailed primer sequences are provided in Table S1.

### Transcriptome sequencing (RNA sequencing)

Total RNA was extracted from cells using a commercial extraction kit. RNA integrity and concentration were determined before library preparation. Samples of appropriate quality were then used for complementary DNA library preparation by poly(A) mRNA enrichment or rRNA depletion. These libraries were sequenced on an Illumina platform using high-throughput sequencing. Raw sequencing reads were subjected to quality filtering to remove adapters and low-quality reads, and the cleaned reads were mapped to the reference genome using appropriate alignment tools. Gene expression levels were quantified and normalized as fragments per kilobase of transcript per million mapped reads or transcripts per million. Differentially expressed genes were identified based on preset thresholds for fold change and adjusted *P* value. Functional annotation and pathway enrichment analyses were performed to elucidate the biological significance of the altered transcripts.

### Nontargeted metabolomics analysis

Cells were washed with cold PBS and quenched with prechilled methanol to arrest metabolic activity. Metabolites were extracted using a methanol–acetonitrile–water mixture, thoroughly vortexed, and clarified by centrifugation to remove precipitated proteins. The supernatant was collected, vacuum-dried, and reconstituted in an appropriate solvent prior to analysis. Metabolic profiling was performed using ultrahigh-performance liquid chromatography coupled with high-resolution mass spectrometry operating in both positive and negative ion modes. Raw spectral data were processed for peak detection, retention time alignment, and signal normalization using dedicated data analysis software. Differential metabolites were identified based on variable importance in projection values, fold change, and statistical significance. Identified metabolites were subjected to pathway enrichment analysis using the KEGG database.

### LC-MS profiling of fatty acid biosynthesis metabolites

To evaluate metabolites associated with fatty acid biosynthesis, cells were rapidly rinsed with ice-cold PBS and quenched with precooled methanol to arrest metabolic activity. Intracellular metabolites were extracted using a methanol–water solvent system, vigorously mixed, and clarified by centrifugation at 14,000 × *g* for 15 min at 4 °C. The supernatant was collected, vacuum-dried, and reconstituted in LC-MS-grade solvent for analysis. Chromatographic separation was performed on a reversed-phase liquid chromatography column, and analytes were detected by mass spectrometry using electrospray ionization. The data were acquired in targeted mode to quantify selected intermediates and end products of fatty acid biosynthetic pathways. Metabolite identification relied on the accurate measurement of mass and retention time, together with comparison to available authentic standards. Relative metabolite abundance was normalized to total protein content or internal standards and statistically analyzed.

### Immunoprecipitation–mass spectrometry

Cell lysates were prepared by using the ice-cold lysis buffer with protease inhibitors and centrifuged to remove cell debris. The supernatants were incubated overnight at 4 °C on a rotating shaker with the antibody directed against the protein of interest. The protein–antibody complexes were captured with Protein A/G agarose or magnetic beads and washed repeatedly to remove nonspecific binding. The bound proteins were eluted, reduced, and alkylated before trypsin digestion into peptides. The peptide mixtures were analyzed by liquid chromatography coupled with tandem mass spectrometry (LC-MS/MS). Database search algorithms were used to search the raw data to identify interacting proteins. Potential binding partners were identified based on enrichment compared with IgG controls and defined statistical criteria.

### Prussian blue staining

To assess intracellular ferric iron levels, Prussian blue staining was performed [10]. Briefly, cells were seeded onto sterile glass coverslips and allowed to adhere overnight. After treatment, the cells were rinsed with distilled water and fixed with 4% paraformaldehyde at room temperature for 15 min. A working staining solution was freshly prepared by combining equal volumes of 2% potassium ferrocyanide and 2% hydrochloric acid. The fixed cells were incubated with this mixture for approximately 20 to 30 min to allow the formation of ferric ferrocyanide precipitates. Following incubation, residual staining solution was removed by gentle washing with distilled water. Cell nuclei were subsequently counterstained with nuclear fast red to improve morphological visualization. Stained samples were examined using a light microscope. The presence of blue granular deposits within the cytoplasm was considered indicative of ferric iron accumulation.

### Immunofluorescence staining

Cells cultured on glass coverslips were fixed in 4% paraformaldehyde at 37 °C for 15 min, permeabilized with 0.1% Triton X-100 at 37 °C for 10 min, followed by blocking in 1% bovine serum albumin for 30 min. Coverslips were incubated with specific primary antibodies overnight at 4 °C. After washing with PBS, coverslips were incubated with fluorescence-conjugated secondary antibodies for 1 h at room temperature in a dark incubator. The nuclear staining was performed with 4′,6-diamidino-2-phenylindole. Images were acquired using a fluorescence microscope.

### Wound-healing migration assay

Cells were plated in 6-well plates and grown until 90% confluence. A straight scratch was made across the monolayer with a sterile 200-μl pipette tip. Detached cells and debris were removed by gentle washing twice with PBS. To minimize the contribution of cell proliferation to wound closure, cells were maintained in a reduced serum-containing medium during migration. Phase-contrast images were taken immediately after scratching (0 h) and at various times using a light microscope. The wound area was measured using ImageJ software, and the percentage of wound closure was calculated relative to the initial wound width.

### Cell cycle analysis

Cell cycle distribution was analyzed by flow cytometry based on DNA content. The cells were trypsinized, washed with PBS, and fixed in 70% ethanol at −20 °C overnight to preserve nuclear DNA. After the ethanol was removed by washing, the cells were incubated with 100 μg/ml ribonuclease A and 50 μg/ml propidium iodide for 30 min at room temperature in the dark to stain the DNA. The samples were analyzed by flow cytometry with excitation at 488 nm, and at least 10,000 events were recorded per sample. The relative percentage of cells in G1, S, and G2/M was estimated using FlowJo software.

### Transmission electron microscopy

The cells were grown in culture in a 4-well chambered cover glass and fixed in 2.5% glutaraldehyde and 2% paraformaldehyde in 0.1 M phosphate buffer at 4 °C for 2 h. Afterward, the samples were washed with phosphate buffer and treated with 1% osmium tetroxide at room temperature for 1 h. This was followed by gradient dehydration with ethanol, embedding in Epon, and section preparation with a diamond knife. Thin sections were mounted on copper grids, stained with uranyl acetate and lead citrate, and analyzed under a transmission electron microscope.

### LLPS assay

To investigate phase separation behavior, cells transfected with EGFP-tagged ZDHHC12 constructs were treated with SCO at the indicated concentrations [52]. The formation of cytoplasmic puncta was monitored by live-cell fluorescence imaging using a confocal microscope. Discrete, spherical EGFP-positive condensates were considered indicative of phase separation behavior. Puncta numbers and spatial distribution were quantified by standardized imaging parameters. Moreover, FRAP analysis was performed to assess the dynamic nature of ZDHHC12 condensates. A single droplet was selectively photobleached by a brief pulse of a high-intensity laser, and fluorescence recovery was monitored over time to assess molecular exchange within the condensates. To probe the liquid-like nature of the assemblies, cells were treated with 1,6-HD, a reagent known to disrupt weak hydrophobic interactions. Dissolution or reduction of puncta following 1,6-HD treatment supported the reversible and dynamic properties of the condensates.

### In vitro LLPS reconstitution assay

Full-length human ZDHHC12 bearing an N-terminal EGFP-affinity tag was cloned into pFastBac1 and expressed in Sf9 cells using the Bac-to-Bac baculovirus system. Recombinant bacmid DNA generated in DH10Bac cells was used to produce baculovirus, and Sf9 cultures were infected at a multiplicity of infection of 5 and harvested 72 h after infection. Cells were lysed in buffer containing 25 mM Hepes, 150 mM NaCl, 1 mM dithiothreitol (DTT), and protease inhibitors. Membrane fractions were collected by ultracentrifugation at 100,000 × *g* for 1 h and extracted with 1% (w/v) n-dodecyl-β-D-maltoside (DDM) for 2 h at 4 °C. After removal of insoluble material by ultracentrifugation, EGFP–ZDHHC12 was purified by affinity chromatography and size-exclusion chromatography using a Superdex 200 Increase column equilibrated with 25 mM Hepes, 150 mM NaCl, 0.03% (w/v) DDM, and 1 mM DTT. Protein purity was assessed by immunoblotting. Freshly purified EGFP–ZDHHC12 was subsequently used for cell-free LLPS assays [52], with the final DDM concentration maintained at 0.03% (w/v) in all reactions. Concentration-dependent condensate formation was examined using 10, 20, 40, and 80 μM EGFP–ZDHHC12. Unless otherwise indicated, 40 μM EGFP–ZDHHC12 was used to evaluate the effects of NaCl concentration (37.5, 150, 300, or 500 mM), pH (6.0, 5.5, 5.0, or 4.5), temperature (4, 16, 25, or 37 °C), and PEG8000 concentration (5%, 8%, 12%, or 15% [w/v]). Each parameter was varied independently while the other reaction components were kept constant. Samples were examined by fluorescence microscopy using identical acquisition settings, and fluorescent droplets were quantified per microscopic field.

### Limited proteolysis assay

Equal amounts of purified ATG12 were incubated with trypsin at a fixed enzyme-to-substrate ratio under identical buffer conditions. Aliquots were collected at 0, 5, 10, 20, and 40 min, and proteolysis was terminated immediately by adding protease inhibitor and SDS sample buffer, followed by heating. The remaining full-length ATG12 was resolved by SDS-PAGE and detected by immunoblotting. Band intensities were quantified using ImageJ, normalized to the corresponding 0-min sample, and fitted to a single-exponential decay model. The apparent proteolysis rate constant 𝑘_obs_ was obtained from the fitted curve, and the half-life was calculated as *t_1/2_* = ln2/𝑘_obs_.

### In vitro palmitoyltransferase assay

Purified recombinant ATG12 was incubated with equal amounts of WT ZDHHC12 or the LLPS-deficient ZDHHC12Δ84–90 mutant in a 25-μl reaction buffer containing 50 mM Hepes, 1 mM DTT, 0.03% (w/v) DDM, and 10 μM palmitoyl-CoA. Matched samples were examined by fluorescence microscopy to verify condensate formation, and reactions containing ATG12 alone served as negative controls. For autoradiographic detection, reactions were supplemented with 1 μCi [^3^H]-palmitoyl-CoA and incubated at 30 °C for 60 min. Reactions were stopped with DTT-containing SDS sample buffer, heated at 95 °C for 5 min, separated by SDS-PAGE, fixed, dried, and analyzed by fluorography to detect [^3^H]-palmitate incorporation into ATG12. For click chemistry-based detection, parallel reactions were performed using 17-ODYA-CoA as a clickable palmitate analog, followed by biotin labeling and streptavidin-based immunoblot detection. Palmitoylated ATG12 signals were quantified by densitometry and normalized to the corresponding total ATG12 loading control. Equivalent enzyme and substrate inputs were maintained in all paired comparisons, and data were obtained from independent replicate reactions.

### Animal experiments

All animal procedures were approved by the Institutional Animal Care and Use Committee of Nanjing University of Chinese Medicine (Approval No. 202509A016). Specific pathogen-free-grade mice (aged 6 to 8 weeks, weighing 18 to 22 g) were acclimatized for 7 d under standard housing conditions before allocation to experimental groups by body-weight-stratified computer randomization. Mice were assigned to experimental groups using a balanced mixed-sex design, with equal numbers of male and female mice in each group. As previously described [34,35], liver fibrosis was established in independent cohorts using CCl_4_ administration and BDL (Fig.S8A and B). For the CCl_4_-induced liver fibrosis model, mice received intraperitoneal injections of CCl_4_ mixed with corn oil at a volume ratio of 1:9 3 times per week from week 0 to week 8, while control mice received an equivalent volume of corn oil. SCO (60 mg/kg) was administered intraperitoneally once daily from week 4 to week 8. VA-Lip-ZDHHC12 KD, VA-Lip-ATG12 KD, or VA-Lip-Control Vector was administered via tail vein injection at weeks 2, 4, and 6. For the cholestatic model, the common bile duct was exposed and ligated under anesthesia at week 0, whereas sham-operated mice underwent the same surgical procedure without ligation. SCO (60 mg/kg) was administered intraperitoneally once daily from week 4 to week 8. VA-Lip-ZDHHC12 KD, VA-Lip-ATG12 KD, or VA-Lip-Control Vector was administered via tail vein injection at weeks 2, 4, and 6. Each model included a normal control group (vehicle or sham), liver fibrosis group (CCl_4_ +VA-Lip-Control Vector or BDL+ VA-Lip-Control Vector), liver fibrosis+SCO+VA-Lip-Control Vector group, liver fibrosis+SCO+VA-Lip-ZDHHC12 KD group, and liver fibrosis+SCO+VA-Lip-ATG12 KD group, with 8 mice per group. Blood samples were collected at the initiation of SCO treatment and weekly thereafter for 4 weeks to dynamically monitor serum biochemical indices. At the experimental endpoint, animals were euthanized, and liver tissues were excised for biochemical and histopathological analyses.

### Histological analysis

Liver tissues were fixed in 4% paraformaldehyde, dehydrated with increasing concentrations of ethanol, and paraffin-embedded. Thin sections of about 4 μm were cut from the paraffin blocks. H&E, Masson’s trichrome, and Sirius Red staining were performed using the automated Leica BOND RX system per the standard protocol. Immunohistochemical staining was performed on sections after deparaffinization, antigen retrieval, and incubation with the specific primary antibodies. Following rinsing, enzyme-linked secondary antibodies were added, and signals were developed using 3,3′-diaminobenzidine as the substrate. The sections were then counterstained and observed under a light microscope.

### Serological analysis

Blood samples were centrifuged, and serum was collected into clean tubes and stored at −80 °C until analysis. Before the measurements, the automated biochemical analyzer was calibrated using standard references and internal quality control samples. Serum samples and assay reagents were loaded into the analyzer, which quantitatively measured the parameters using the respective colorimetric detection methods. The concentration of each biomarker was then automatically calculated based on the manufacturer’s calibration data.

### Isolation and validation of primary liver cells

Primary HSCs, hepatocytes, and macrophages were isolated from freshly harvested livers of CCl_4_-treated mice in each experimental group [34,35]. After anesthesia, the liver was perfused through the portal vein with Ca^2+^/Mg^2+^-free Hank’s balanced salt solution containing 0.5 mM EGTA, followed by digestion with 0.05% collagenase IV at 37 °C. The dissociated tissue was filtered through a 70-μm cell strainer and subjected to differential centrifugation. Hepatocytes were collected by repeated low-speed centrifugation (50 × *g*, 3 min) and purified using a Percoll gradient. The nonparenchymal fraction was further separated by OptiPrep density-gradient centrifugation. HSCs were recovered from the low-density interface, whereas macrophages were enriched from the remaining fraction using F4/80 magnetic beads. The different cell types were further characterized by morphology and specific marker expression: desmin and α-SMA for HSCs, albumin and HNF4α for hepatocytes, and F4/80 and CD68 for macrophages.

### Bioinformatic analysis of human liver fibrosis transcriptomes

Public human liver transcriptomics data were obtained from the Gene Expression Omnibus database. The single-cell RNA-sequencing dataset GSE136103 was used to examine the cell type-specific distribution of ZDHHC12 and ATG12. This dataset was generated using the Illumina HiSeq 4000 platform (GPL20301) and contains transcriptomic profiles of more than 100,000 single cells derived from 5 healthy and 5 fibrotic human livers. Processed expression matrices and metadata were analyzed using Seurat. After quality control, normalization, identification of highly variable genes, principal component analysis, and batch correction, cells were clustered by uniform manifold approximation and projection and annotated using canonical markers. Feature plots were generated to visualize ZDHHC12 and ATG12 expression across hepatic cell populations. Pseudotime analysis of HSCs was performed using Monocle 3 to assess changes in gene expression along the inferred activation trajectory. The bulk liver transcriptomic dataset GSE84044 was used to evaluate the associations of ZDHHC12 and ATG12 expression with fibrosis stage. This dataset comprises liver biopsy specimens from 124 patients with chronic hepatitis B and was generated using the Affymetrix Human Genome U133 Plus 2.0 Array platform (GPL570). Expression differences across fibrosis stages were analyzed and visualized using violin plots. Correlation analysis between ZDHHC12 and ATG12 expression was performed in R using Pearson correlation coefficients, and statistical significance was calculated. The ClusterProfiler package (v4.6.2) was used to perform gene set enrichment analysis, which allows the exploration of relationships between ZDHHC12 or ATG12 expression and gene sets related to hepatic fibrosis and ferroptosis. Normalized enrichment scores and adjusted *P* values were used to evaluate the significance and direction of pathway enrichment. Patients were divided into high- and low-expression groups according to the median expression level of each hub gene. Fibrosis progression-free survival was defined as the time from baseline sampling to documented fibrosis progression, whereas cirrhosis-free survival was defined as the time from baseline sampling to clinically diagnosed cirrhosis during follow-up. Kaplan–Meier curves were generated to compare progression-free survival between the high- and low-expression groups, and differences were assessed using the log-rank test. The original raw survival data used for Kaplan–Meier analyses are provided in Table S2. Multivariate Cox proportional hazards regression was performed to estimate hazard ratios and 95% confidence intervals for fibrosis progression after adjustment for relevant clinical covariates. The results were visualized using a forest plot. Receiver operating characteristic curve analysis was performed to evaluate the discriminative ability of hub gene expression levels for identifying advanced fibrosis. Advanced fibrosis was defined as fibrosis stages F3 to F4, whereas F0 to F2 were defined as nonadvanced fibrosis. The area under the curve was calculated.

### Human liver fibrosis cohort and tissue analysis

Fibrotic and relatively nonfibrotic liver tissues were obtained from 40 adult patients undergoing diagnostic biopsy or surgical resection at the affiliated hospital. The study was conducted with institutional ethics approval and written informed consent from all participants. Inclusion criteria were as follows: (a) age ≥18 years; (b) histologically confirmed liver fibrosis based on liver biopsy or resection specimens, with staging according to the METAVIR scoring system; (c) availability of fibrotic and matched relatively nonfibrotic liver tissues; and (d) complete basic clinicopathological data. Exclusion criteria included: (a) presence of primary or metastatic liver malignancy; (b) coexistence of other severe systemic diseases; (c) prior antifibrotic, chemotherapy, or immunosuppressive therapy within 6 months before sample collection; and (d) inadequate tissue quality for histological or protein analysis. Detailed clinicopathological characteristics are summarized in Table S3. Fibrotic tissues were collected from regions with evident collagen deposition and fibrotic septa, whereas relatively nonfibrotic tissues were obtained from nonlesional liver regions and were histologically confirmed to lack significant fibrotic deposition by H&E and Masson’s trichrome staining. The expression of ZDHHC12 and ATG12 was determined in tissues by western blot and RT-qPCR. The relationship between ZDHHC12 and ATG12 expression was evaluated using Pearson correlation analysis. The expression of ZDHHC12 and ATG12 in liver tissues classified as METAVIR F1 to F4 was determined by western blot and RT-qPCR. To evaluate trends in gene expression across increasing fibrosis severity, linear trend analysis was performed using simple linear regression. Serum ALT and AST values obtained at sample collection were retrieved from clinical records. The relationships between ZDHHC12 or ATG12 expression and serum ALT or AST levels were examined using Pearson’s correlation analysis, followed by linear regression modeling. To identify variables independently associated with advanced fibrosis (METAVIR stages F3 to F4), multivariable logistic regression analysis was conducted. Effect estimates were presented as odds ratios with corresponding 95% confidence intervals.

### Isolation and characterization of primary hHSCs

Primary hHSCs were isolated from freshly resected liver tissues obtained from at least 3 independent donors [34,35]. Samples were transported in ice-cold Hank’s balanced salt solution and processed within 2 h of collection. After carefully excising visible blood vessels and connective tissue, liver samples were finely chopped into fragments of approximately 1 mm^3^. The tissue pieces were incubated at 37 °C for 30 min in granule-bound starch synthase supplemented with 0.02% (w/v) pronase, 0.05% (w/v) collagenase IV, and 20 μg/ml deoxyribonuclease I under gentle agitation to facilitate enzymatic dissociation. Enzymatic activity was quenched by adding DMEM containing 10% FBS. The resulting cell suspension was filtered through a 70-μm mesh to remove undigested debris. To separate parenchymal and nonparenchymal fractions, hepatocytes were pelleted by low-speed centrifugation at 50 × *g* for 7 min at 4 °C. The supernatant was subsequently centrifuged at 450 × *g* for 8 min to collect nonparenchymal cells. The cell pellet was resuspended in 11% (w/v) Nycodenz solution, carefully overlaid with granule-bound starch synthase, and centrifuged at 2,000 × *g* for 20 min at 4 °C without brake. The interface corresponding to the low-density HSC-enriched fraction was aspirated, washed, and maintained in DMEM supplemented with 10% FBS and 1% penicillin–streptomycin at 37 °C in a humidified incubator containing 5% CO₂. HSC identity was verified based on VA autofluorescence and immunofluorescent detection of desmin, vimentin, and platelet-derived growth factor receptor β.

### Statistical analysis

Data are expressed as mean ± standard error of the mean. Comparisons between 2 independent groups were performed using an unpaired 2-tailed Student *t* test, whereas fibrotic and nonfibrotic tissues from the same patient were compared using a paired *t* test. One-way analysis of variance (ANOVA), followed by Tukey’s multiple-comparison test, was used for comparisons involving more than 2 groups with a single factor. Factorial experiments involving treatment and pharmacological or genetic intervention were analyzed using 2-way ANOVA, including assessment of the interaction between the 2 factors, followed by Bonferroni-adjusted pairwise comparisons where appropriate. Two-way repeated-measures ANOVA with Bonferroni-adjusted pairwise comparisons was used for longitudinal measurements. Pearson’s correlation was used for normally distributed continuous variables, whereas Spearman’s correlation was used for fibrosis stages or non-normally distributed variables. All statistical evaluations were conducted using GraphPad Prism version 8.0. Differences were considered statistically significant at *P* < 0.05.

## Ethical Approval

All animal experimental protocols complied with institutional guidelines and were approved by the Institutional Animal Care and Use Committee of Nanjing University of Chinese Medicine. All procedures involving human samples were approved by the Ethics Committee of Nanjing University of Chinese Medicine and adhered to the principles of the Declaration of Helsinki.

## Data Availability

The datasets used and analyzed in this study will be made available from the corresponding author upon reasonable request.
