# Supplementary material for: Scoparone-Induced Phase Separation of ZDHHC12 Drives Ferroptosis via ATG12 S-palmitoylation and Ferritinophagy in Liver Fibrosis
Source: Research (Wash D C). 2026 Sep 30;9:1450. doi: 10.34133/research.1450 (PMC13624940; doi:10.34133/research.1450)
Supplement: Supplementary file 1 [file research.1450.f1.zip › Supplementary Figure Legends.docx]

**Supplementary Figure Legends**

**Figure S1: SCO inhibits the activation of human HSCs by inducing ferroptosis through ZDHHC12-mediated ATG12 S-palmitoylation.** Primary human HSCs were treated with SCO (5, 10, 20 μM) for 24 hours. **(A)** Cell proliferation was assessed by the EdU assay (n = 3, ^*^P < 0.05, ^**^P < 0.01, ^***^P < 0.001). **(B)** The expression of α-SMA, collagen I, and fibronectin was determined by western blot and RT-qPCR (n = 3, ^*^P < 0.05, ^**^P < 0.01, ^***^P < 0.001). **(C)** The intracellular iron, lipid ROS, MDA, and GSH levels were examined by commercial assay kits (n = 3, ^*^P < 0.05, ^**^P < 0.01, ^***^P < 0.001). Primary human HSCs were treated with SCO (20 μM) and ferrostatin-1 (Fer-1, 10 μM) for 24 hours. **(D)** The intracellular iron, lipid ROS, MDA, and GSH levels were examined by commercial assay kits (n = 3, ^*^P < 0.05, ^**^P < 0.01). **(E)** Cell proliferation was assessed by the EdU assay (n = 3, ^**^P < 0.01). **(F)** The expression of α-SMA, collagen I, and fibronectin was determined by western blot and RT-qPCR (n = 3, ^*^P < 0.05, ^**^P < 0.01). **(G)** The expression of ZDHHC12, ATG12, LC3-I/II, and p62 was determined by western blotting (n = 3, ^*^P < 0.05, ^**^P < 0.01, ^***^P < 0.001). **(H)** The ATG12 S-palmitoylation was examined by the acyl‑biotin exchange assay (n = 3, ^*^P < 0.05, ^**^P < 0.01, ^***^P < 0.001). **(I)** Primary human HSCs were exposed to SCO (20 μM) for 24 hours. Fluorescence microscopy images of EGFP‑tagged ZDHHC12 were shown. Scale bar: 10 μm (n = 3). **(J)** Rescue experiments were performed by re-expressing ATG12 WT or ATG12 C134A mutant in ATG12 KD cells, followed by treatment with SCO (20 μM) for 24 hours. The intracellular iron and MDA levels were detected by commercial assay kits (n = 3, ^*^P < 0.05, ^**^P < 0.01, ^***^P < 0.001, N.S., not significant). **(K)** The expression of *ACTA2*, *COL1A1*, and *FN1* was determined by RT-qPCR (n = 3, ^*^P < 0.05, ^**^P < 0.01, N.S., not significant).

**Figure S2: SCO suppresses HSC activation via ferroptosis induction in HSC-LX2 cells.** HSC-LX2 cells were treated with SCO (5, 10, 20 μM) or Silymarin (SM, 20 μM) for 24 hours. **(A)** Representative images of EdU incorporation assay were shown. Bar: 50 μm (n = 3). **(B)** Cell viability was assessed by CCK‑8 assay (n = 3). **(C)** HSC‑LX2 cells were stimulated with TGF‑β1 (5 ng/mL) for 6 h, followed by treatment with SCO (5, 10, or 20 μM) for 24 hours. The expression of *ACTA2*, *COL1A1*, and *FN1* was determined by RT-qPCR (n = 3, ^*^P < 0.05, ^**^P < 0.01, ^***^P < 0.001). **(D)** Quantitative analysis of wound healing percentage was shown (n = 3, ^*^P < 0.05, ^**^P < 0.01). **(E)** Cell cycle distribution was quantified (n = 3, ^*^P < 0.05, ^**^P < 0.01). **(F)** HSC-LX2 cells were treated with SCO (20 μM) and ferrostatin-1 (Fer-1, 10 μM) for 24 hours. Representative images of EdU incorporation assay were shown. Bar: 50 μm (n = 3).

**Figure S3:** **SCO promotes S-palmitoylation to induce HSC ferroptosis.** HSC-LX2 cells were treated with SCO (5, 10, 20 μM) for 24 hours. **(A)** Quantification of ^13^C incorporation into palmitic acid was analyzed (n = 3, ^*^P < 0.05, ^**^P < 0.01, ^***^P < 0.001). **(B)** The expression of SREBP1, ACC1, and FASN was determined by western blot and RT-qPCR (n = 3, ^*^P < 0.05, ^**^P < 0.01, ^***^P < 0.001). **(C)** HSC-LX2 cells were treated with SCO (20 μM) and fatostatin (10 μM) for 24 hours. Quantification of palmitic acid levels was performed by LC–MS analysis (n = 3, ^**^P < 0.01). **(D)** HSC-LX2 cells were treated with SCO (20 μM) and 2-BP (10 μM) or Palm B (10 μM) for 24 hours. The intracellular iron and MDA levels were examined by commercial assay kits (n = 3, ^*^P < 0.05, ^**^P < 0.01). **(E)** HSC-LX2 cells were treated with SCO (5, 10, 20 μM) for 24 hours. The expression of PPARγ, p-AMPK, AMPK, p-mTOR, and mTOR was determined by western blot (n = 3, ^*^P < 0.05, ^**^P < 0.01, ^***^P < 0.001). **(F)** HSC-LX2 cells were treated with SCO (20 μM) and rapamycin (100 nM) for 24 hours. Quantification of palmitic acid levels was performed by LC–MS analysis. The expression of ZDHHC12 was determined by western blot (n = 3, ^**^P < 0.01). **(G)** HSC-LX2 cells stably transfected with ZDHHC12 shRNA (KD) were exposed to SCO (20 μM) for 24 hours. Transfection efficiency was validated by western blot and RT-qPCR (n = 3, ^***^P < 0.001). **(H)** The intracellular iron, lipid ROS, MDA, and GSH levels were examined by commercial assay kits (n = 3, ^**^P < 0.01). **(I)** Representative images of EdU incorporation assay were shown. Bar: 50 μm (n = 3).

**Figure S4: SCO induces HSC ferroptosis by regulating S-palmitoylation-mediated autophagy. (A, B)** Heatmap of differentially expressed genes was identified by transcriptome sequencing in wild‑type (WT) and ZDHHC12 knockdown (KD) HSC-LX2 cells. KEGG pathway enrichment analysis of differentially expressed genes was shown (n = 3, |log_2_FC| > 1, adjusted P < 0.05). **(C)** GO enrichment analysis was shown (n = 3). HSC-LX2 cells stably transfected with ZDHHC12 shRNA (KD) or ZDHHC12 overexpression (OE) plasmid were exposed to SCO (20 μM) for 24 hours. **(D)** FTH1 protein expression was quantified (n = 3, ^**^P < 0.01). **(E)** The staining of ATG12 and LC3 was examined by immunofluorescence. Bar: 10 μm (n = 3). **(F)** The protein degradation percentage was detected by mass spectrometry (n = 3, ^**^P < 0.01).

**Figure S5: ZDHHC12 promotes ATG12 S-palmitoylation at Cys134 to induce ferroptosis in SCO-treated HSCs.** HSC-LX2 cells stably expressing ATG12 WT or ATG12 C134A mutant were exposed to SCO (20 μM) for 24 hours. **(A)** ATG12 protein expression was quantified (n = 3, ^*^P < 0.05, ^**^P < 0.01, N.S., not significant). **(B)** ATG12 S-palmitoylation levels were quantified (n = 3, ^*^P < 0.05, ^**^P < 0.01, N.S., not significant). **(C)** The LC3‑II/LC3‑I ratio and p62 protein levels were quantified (n = 3, ^*^P < 0.05, ^**^P < 0.01, N.S., not significant). **(D)** Rescue experiments were performed by re-expressing ZDHHC12 in ZDHHC12 KD cells, followed by treatment with SCO (20 μM) for 24 hours. ZDHHC12 and ATG12 protein expression were quantified (n = 3, ^**^P < 0.01, N.S., not significant).

**Figure S6: S-palmitoylation enhances ATG12 structural stability. (A)** Limited proteolysis assay comparing ATG12 WT and Cys134‑palmitoylated ATG12 following trypsin digestion for the indicated times (0–40 min) (n = 3). **(B)** Quantification of relative full‑length ATG12 intensity during trypsin digestion was shown (n = 3, ^*^P < 0.05). **(C)** Apparent degradation rate constant (*k_obs_*) and half‑life (*t_1/2_*) of ATG12 WT and Cys134‑palmitoylated ATG12 were calculated based on proteolysis kinetics (n = 3, ^*^P < 0.05). **(D)** Thermal stability analysis of ATG12 WT and Cys134‑palmitoylated ATG12 was shown. Fluorescence ratio curves were plotted against temperature, and the apparent melting temperature (T_m_) was determined (n = 3, ^*^P < 0.05). **(E)** HSC-LX2 cells stably expressing ATG12 WT or ATG12 C134A mutant were exposed to SCO (20 μM) for 24 hours. The interaction between ATG12 and ATG5 was determined by Co‑immunoprecipitation assays (n = 3). **(F)** ATG12 ubiquitination levels were assessed by immunoprecipitation followed by immunoblotting (n = 3). ATG12 protein levels were assessed by immunoblotting (n = 3).

**Figure S7: ZDHHC12 undergoes phase separation under acidic and stress conditions. (A)** Cell‑free in vitro liquid–liquid phase separation (LLPS) assay was used to show droplet formation of EGFP‑ZDHHC12 under different pH conditions (pH 6.0, 5.5, 5.0, and 4.5) (n = 3, ^*^P < 0.05, ^**^P < 0.01, ^***^P < 0.001 compared with pH 6.0 group). **(B)** LLPS analysis of EGFP‑ZDHHC12 at different temperatures (4°C, 16°C, 25°C, and 37°C) was shown (n = 3, ^*^P < 0.05, ^***^P < 0.001 compared with 4°C group). **(C)** LLPS assay of EGFP‑ZDHHC12 in the presence of increasing concentrations of PEG8000 (5%, 8%, 12%, and 15%, w/v) was shown (n = 3, ^*^P < 0.05, ^**^P < 0.01, ^***^P < 0.001 compared with 5% group). **(D)** Palmitoylation activity was assessed by autoradiography assay using [^3^H]-palmitoyl‑CoA in cells expressing ZDHHC12 WT or ZDHHC12Δ84–90 mutant (n = 3, ^***^P < 0.001). **(E)** Click chemistry assay was used to determine ATG12 S-palmitoylation in ZDHHC12 WT or ZDHHC12Δ84–90 mutant groups (n = 3, ^**^P < 0.01). **(F)** HSC-LX2 cells stably expressing ZDHHC12 WT or ZDHHC12Δ84–90 were exposed to SCO (20 μM) for 24 hours. ZDHHC12 protein expression was quantified (n = 3, ^*^P < 0.05, N.S., not significant). **(G)** ATG12 palmitoylation levels were quantified (n = 3, ^*^P < 0.05, ^**^P < 0.01, N.S., not significant). **(H)** The LC3‑II/LC3‑I ratio, ATG12, and p62 protein levels were quantified (n = 3, ^*^P < 0.05, ^**^P < 0.01, N.S., not significant).

**Figure S8: SCO alleviates liver fibrosis by inducing HSC ferroptosis through ZDHHC12-mediated ATG12 S-palmitoylation in cholestatic fibrosis. (A)** Schematic diagram of the CCl_4_‑induced liver fibrosis model and treatment schedule were shown. **(B)** Schematic illustration of the bile duct ligation model and experimental design were shown. **(C)** Representative gross morphology of liver tissues. Scale bar: 1 cm (n = 8). **(D)** H&E, Sirius Red, Masson, and α‑SMA immunohistochemical staining were performed to assess collagen deposition. Scale bar: 10 μm (n = 8). **(E)** Serum ALT levels were detected by serum biochemical analysis (n = 8, statistical comparisons were performed among groups at the same time point. ^***^P < 0.001 compared with the Sham group, ^#^P < 0.05, ^##^P < 0.01, ^###^P < 0.001 compared with the BDL+VA‑Lip‑Control Vector group, ^&^P < 0.05 compared with the BDL+SCO+VA‑Lip‑Control Vector group). **(F)** ATG12 S-palmitoylation levels in primary HSCs were determined by acyl‑biotin exchange assay (n = 8). **(G)** The expression of ZDHHC12, ATG12, LC3-I/II, and p62 was determined by western blotting (n = 8). **(H)** Immunohistochemical staining for 4‑HNE and Prussian blue staining were used to evaluate lipid peroxidation and iron deposition. Scale bar: 10 μm (n = 8). **(I)** The iron and MDA levels were examined by commercial assay kits (n = 8, ^##^P < 0.01 compared with the BDL+VA‑Lip‑ Control Vector group, ^&^P < 0.05 compared with the BDL+SCO+VA‑Lip‑Control Vector group).

**Figure S9: The effects of SCO on ZDHHC12 expression, ATG12 expression, and ATG12 S-palmitoylation in primary HSCs, hepatocytes, and macrophages. (A)** The expression of collagen I, TIMP1, and α‑SMA in liver tissues was determined by western blotting (n = 8, ^**^P < 0.01, ^***^P < 0.001 compared with the Vehicle group, ^#^P < 0.05, ^##^P < 0.01 compared with the CCl_4_+VA‑Lip‑Control Vector group, ^&^P < 0.05, ^&&^P < 0.01 compared with the CCl_4_+SCO+VA‑Lip‑Control Vector group). **(B)** Serum AST and TGF-β1 levels were detected (n = 8, statistical comparisons were performed among groups at the same time point. ^***^P < 0.001 compared with the Vehicle group, ^#^P < 0.05, ^##^P < 0.01, ^###^P < 0.001 compared with the CCl_4_+VA‑Lip‑Control Vector group, ^&^P < 0.05, ^&&^P < 0.01 compared with the CCl_4_+SCO+VA‑Lip‑Control Vector group). **(C)** ZDHHC12 and ATG12 expression in healthy and CCl_4_-induced fibrotic liver tissues was analyzed by western blot and RT-qPCR (n = 8, ^*^P < 0.05, ^**^P < 0.01 compared with healthy group). **(D)** ATG12 S-palmitoylation levels in primary hepatocyte and macrophage were determined by acyl‑biotin exchange assay (n = 8, ^##^P < 0.01 compared with the CCl_4_+VA‑Lip‑Control Vector group, ^&^P < 0.05 compared with the CCl_4_+SCO+VA‑Lip‑Control Vector group). **(E, F)** The expression of ZDHHC12, ATG12, LC3-I/II, and p62 in primary hepatocyte and macrophage was determined by western blotting (n = 8, ^*^P < 0.05 compared with the Vehicle group, ^#^P < 0.05, ^##^P < 0.01 compared with the CCl_4_+VA‑Lip‑Control Vector group, ^&^P < 0.05, ^&&^P < 0.01 compared with the CCl_4_+SCO+VA‑Lip‑Control Vector group).

**Figure S10: Clinical relevance of ZDHHC12 and ATG12 expression in human liver fibrosis. (A)** ZDHHC12 and ATG12 expression levels in paired fibrotic and adjacent non‑fibrotic human liver tissues were analyzed by western blot and RT‑qPCR (n = 40, ^**^P < 0.01). **(B)** The association between ZDHHC12 and ATG12 expression levels in fibrotic specimens was determined by Pearson correlation analysis (n = 40, R = 0.62, P < 0.001). **(C)** ZDHHC12 and ATG12 expression across different fibrosis stages was determined by western blot and RT-qPCR (n = 10 per group, ^*^P < 0.05, ^**^P < 0.01, ^***^P < 0.001). The trend of ZDHHC12 and ATG12 expression across fibrosis stages was examined using linear trend analysis. **(D)** Correlation analyses between ZDHHC12 or ATG12 expression and serum ALT or AST levels were performed using Pearson correlation analysis. Pearson correlation coefficients (R) and corresponding P values were indicated. **(E)** Multivariate logistic regression analysis was performed to identify factors independently associated with advanced fibrosis (F3–F4). Odds ratios (ORs) with 95% confidence intervals (CIs) and corresponding P values were shown.
