# Supplementary material for: Scoparone-Induced Phase Separation of ZDHHC12 Drives Ferroptosis via ATG12 S-palmitoylation and Ferritinophagy in Liver Fibrosis
Source: Research (Wash D C). 2026 Sep 30;9:1450. doi: 10.34133/research.1450 (PMC13624940; doi:10.34133/research.1450)
Supplement: Supplementary file 1 [file research.1450.f1.zip › Table S1.docx]

**Table S1. Primer sequences for indicated genes**

| **Genes** | **Forward primer** | **Reverse primer** | **Species** |
| --- | --- | --- | --- |
| *ACTA2* | 5′-CGCTGTCAGGAACCCTGAGA-3′ | 5′-CGAAGCCGGCCTTACAGA-3′ | Human |
| *COL1A1* | 5′-GCTCCTCTTAGGGGCCACT-3′ | 5′-CCACGTCTCACCATTGGGG-3′ | Human |
| *FN1* | 5′-GGCCACACCTACAACCAGTA-3′ | 5′-TCGTCTCTGTCAGCTTGCAC-3′ | Human |
| *GPX4* | 5′-GAAGCAGGAGCCAGGGAGTA-3′ | 5′-GGTGAAGTTCCACTTGATGGC-3′ | Human |
| *SLC7A11* | 5′-TGCTGGGCTGATTTTATCTTCG-3′ | 5′-GAAAGGGCAACCATGAAGAGG-3′ | Human |
| *ACSL4* | 5′-GTTTACGAAGTGAAGCTCAAC-3′ | 5′-GTCTCATCAATCATGTTCGCG-3′ | Human |
| *ZDHHC1* | 5′-CAACTTGTGCAACGTGGATGT-3′ | 5′-AAGAGCCGGTAGTTCCGCT-3′ | Human |
| *ZDHHC2* | 5′-CTCGCTTCTCAGCCCTTGAT-3′ | 5′-TCACATACTTCAGGGCACCG-3′ | Human |
| *ZDHHC3* | 5′-ATGATGCTTATCCCCACC-3′ | 5′-TCAGACCACATACTGGTA-3′ | Human |
| *ZDHHC4* | 5′-TGTCTCGTCAAGCACCATCC-3′ | 5′-CTTGAAGTCAGAAACCTACAGCA-3′ | Human |
| *ZDHHC5* | 5′-ACACCTCGGCTTGGCTACTA-3′ | 5′-GTTGGCTCCTTCAAGCTGTC-3′ | Human |
| *ZDHHC6* | 5′-GTGCCTTGTAGCGTGTGGAT-3′ | 5′-TCGTCGCAATCTCGGTTCTC-3′ | Human |
| *ZDHHC7* | 5′-GAGAACCATGCTCACTGACC-3′ | 5′-GCACTTGTAGATCACCTCGC-3′ | Human |
| *ZDHHC8* | 5′-CTTGGCTGGGCACTCATAGG-3′ | 5′-CCCCAAAGAGAAAGCCCCAT-3′ | Human |
| *ZDHHC9* | 5′-GTCTTCGCCTTCAACATCGT-3′ | 5′-AATCCAGTCAGTCCCACGAC-3′ | Human |
| *ZDHHC11* | 5′-GGTGGCTTTTACCCCAACCT-3′ | 5′-AATGGCCTCTGTCGGTGATG-3′ | Human |
| *ZDHHC12* | 5′-TTGGTTCCATGAGTGGCTGT-3′ | 5′-AAGTCCAGGCAAACCCTCAG-3′ | Human |
| *ZDHHC13* | 5′-CTGTGCTGTTCTGGCTGGT-3′ | 5′-GCATGTGAATGAGCAGAGG-3′ | Human |
| *ZDHHC14* | 5′-ATCACCACTGTCCCTGGGTA-3′ | 5′-AGGAATCCTGTTTGCTGTGAA-3′ | Human |
| *ZDHHC15* | 5′-ATCAAATGTCCCCGTGCCTT-3′ | 5′-GCTGTCTGGAATCTGCCTGT-3′ | Human |
| *ZDHHC16* | 5′-CCACCAGACTCCACCACCTACC-3′ | 5′-GCCACAGAACTGCACAGGAACC-3′ | Human |
| *ZDHHC17* | 5′-TGGATCAACTTGGAGGGGAC-3′ | 5′-TTTTGCTTGCCTTGCCTCTT-3′ | Human |
| *ZDHHC18* | 5′-TGCTGCTGTTCTTCGTCATG-3′ | 5′-AGCAGGATGATGACGATGACC-3′ | Human |
| *ZDHHC19* | 5′-TTGCTGCCTTCAATGCGGTG-3′ | 5′-CGGAGCCTTGATGTAAGATGC-3′ | Human |
| *ZDHHC20* | 5′-CGGTGGGTGGTCAAGAAGTA-3′ | 5′-TGTCAGCTCAGAAGTTGGTG-3′ | Human |
| *ZDHHC21* | 5′-AAGCGTTCCCATCACTGCAGC-3′ | 5′-GAACTCGCAGTGGTTGCCTCTG-3′ | Human |
| *ZDHHC22* | 5′-TGAGTGTTGCCTGGGGTTTT-3′ | 5′-CGATTCCTTAGCCCCTGGAA-3′ | Human |
| *ZDHHC23* | 5′-GTCGGGCAGTCTCAACAATC-3′ | 5′-TCCTCACACAGATGCCACAT-3′ | Human |
| *ZDHHC24* | 5′-CGCTGCACTTGTCTCAACAC-3′ | 5′-GAAGGGCCTGGGACATCTTG-3′ | Human |
| *LYPLA1/APT1* | 5′-AAGGCCTACAGGTGCAGTTC-3′ | 5′-CCAGATTGTTGCAGCGGTTC-3′ | Human |
| *LYPLA2/APT2* | 5′-AAGAAGGCAGCAGAGAACATC-3′ | 5′-CTCCCAGCACGATTCGATTG-3′ | Human |
| *ATG3* | 5′-ATTGCGACAGTCTCTCCGTG-3′ | 5′-ACACCGCTTGTAGCATGGAA-3′ | Human |
| *ATG4B* | 5′-TGGTCAGATGGTTCTCGCC-3′ | 5′-TTCAAGGCAGCGCTTTAAGG-3′ | Human |
| *ATG5* | 5′-TTGACGTTGGTAACTGACAAAGT-3′ | 5′-TGTGATGTTCCAAGGAAGAGC-3′ | Human |
| *BECN1* | 5′-CCATGCAGGTGAGCTTCGT-3′ | 5′-GAATCTGCGAGAGACACCATC-3′ | Human |
| *ATG7* | 5′-TAGCCCAGATTGTCCTAAAG-3′ | 5′-CAAATTCATACAGAGGCTTGC-3′ | Human |
| *MAP1LC3A* | 5′-AGCTTCGCCGACCGCTGTAAG-3′ | 5′-CTTCTCCTGTTCATAGATGTCAGC-3′ | Human |
| *ATG9A* | 5′-TGTTTCTCAATGAATGGAGCCTC-3′ | 5′-AAGTTAGCGATGCCAATCCAC-3′ | Human |
| *ATG10* | 5′-CAGCGGTGGCAGAAGTGATT-3′ | 5′-TGTTCCTGCTGGGTGATGGT-3′ | Human |
| *ATG12* | 5′-AATCATGCAGGGACACAGTT-3′ | 5′-CACTCTTCCTGCTCAGGTGT-3′ | Human |
| *ATG13* | 5′-GCGGAGTCTTAGGAGCAAAA-3′ | 5′-GGTTCCGGGCTCTCATTAC-3′ | Human |
| *ATG14* | 5′-TGCATTGGGAAAGCTGCAAC-3′ | 5′-TCTGTGTGATCAACCTGACCTG-3′ | Human |
| *ATG16L1* | 5′-CAGTTACGTGGCGGCAGGCT-3′ | 5′-ACAACGTGCGAGCCAGAGGG-3′ | Human |
| *SQSTM1* | 5′-AGCTGCCCTTAGCCCTCTG-3′ | 5′-GGCTTCTTTTCCCTCCGTGCT-3′ | Human |
| *NCOA4* | 5′-ATTGAAGAAATTGCAGGCTC-3′ | 5′-TGGAGAAGAGGAGCTGTATCT-3′ | Human |
| *FTH1* | 5′-AATTTCTTGACCCACTGGTGCACT-3′ | 5′-TCGAATCGAGAGTAGTGGCACA-3′ | Human |
| *PIK3CA* | 5′-CGCTTGATGGATTTACTCTGGA-3′ | 5′-GAGGTGCTCACAACTTCAATG-3′ | Human |
| *AKT1* | 5′-GTGCTGGAGGACAACGACT-3′ | 5′-GTGTAGGGTCCTTCTTGAGCA-3′ | Human |
| *MTOR* | 5′-CCCAGCTGCTGGAACAAAAA-3′ | 5′-GTCATGCCCACGTTCCTTAAC-3′ | Human |
| *ULK1* | 5′-AAGCACGATTTGGAGGTCGC-3′ | 5′-TGATTTCCTTCCCCAGCAGC-3′ | Human |
| *SREBP1* | 5′-GCAGCCACCATCTAGCCTG-3′ | 5′-CAGCAGTGAGTCTGCCTTGAT-3′ | Human |
| *ACACA* | 5′-ATGGGCGGAATGGTCTCTTTC-3′ | 5′-TGGGGACCTTGTCTTCATCAT-3′ | Human |
| *FASN* | 5′-CACAGGGACAACCTGGAGTT-3′ | 5′-ACTCCACAGGTGGGAACAAG-3′ | Human |
| *GAPDH* | 5′-TGTGAGGGAGATGCTCAGTG-3′ | 5′-TGTTCCTACCCCCAATGTGT-3′ | Human |
| *Atg12* | 5′-TCCGTGCCATCACATACACA-3′ | 5′-TAAGACTGCTGTGGGGCTGA-3′ | Mouse |
| *Zdhhc12* | 5′-CTGTGTGGGTGAACGCAAC-3′ | 5′-CACTAACGCGAAGAAGGAGAG-3′ | Mouse |
| *Ptgs2* | 5′-TCCAACCTCTCCTACTACACCAG-3′ | 5′-GGGTCAGGGATGAACTCTCTC-3′ | Mouse |
| *Gapdh* | 5′-TGGCAAAGTGGAGATTGTTG-3′ | 5′-CATTATCGGCCTTGACTGTG-3′ | Mouse |
