# Supplementary material for: Scoparone-Induced Phase Separation of ZDHHC12 Drives Ferroptosis via ATG12 S-palmitoylation and Ferritinophagy in Liver Fibrosis
Source: Research (Wash D C). 2026 Sep 30;9:1450. doi: 10.34133/research.1450 (PMC13624940; doi:10.34133/research.1450)
Supplement: Supplementary file 1 [file research.1450.f1.zip › Table S2.docx]

**Table S2. Raw survival data for Kaplan–Meier analyses**

| **Patient ID** | **ZDHHC12 group** | **ATG12 group** | **PFS time (months)** | **Fibrosis progression event** | **Cirrhosis free time (months)** | **Cirrhosis event** | **Age (years)** | **Sex** | **Baseline fibrosis stage** | **Etiology** |
| --- | --- | --- | --- | --- | --- | --- | --- | --- | --- | --- |
| P001 | High | High | 5.2 | 0 | 6.1 | 0 | 36 | Male | F1 | HBV |
| P002 | Low | Low | 7.4 | 1 | 9.2 | 1 | 58 | Male | F3 | HBV |
| P003 | Low | Low | 11.8 | 0 | 13.5 | 0 | 43 | Female | F2 | HBV |
| P004 | High | High | 13.6 | 1 | 16.4 | 0 | 49 | Male | F2 | HBV |
| P005 | High | High | 15.9 | 0 | 18.7 | 0 | 39 | Female | F1 | HBV |
| P006 | Low | Low | 18.2 | 1 | 20.6 | 1 | 61 | Male | F3 | HBV |
| P007 | High | High | 20.7 | 1 | 23.9 | 0 | 52 | Male | F2 | HBV |
| P008 | Low | Low | 22.5 | 1 | 25.4 | 0 | 55 | Female | F2 | HBV |
| P009 | High | Low | 24.8 | 0 | 28.2 | 0 | 41 | Female | F1 | HBV |
| P010 | Low | High | 27.1 | 1 | 30.5 | 0 | 47 | Male | F2 | HBV |
| P011 | High | High | 29.4 | 0 | 33.1 | 0 | 38 | Male | F1 | HBV |
| P012 | Low | Low | 31.6 | 1 | 35.7 | 1 | 59 | Male | F3 | HBV |
| P013 | High | High | 34.2 | 0 | 38.4 | 0 | 45 | Female | F2 | HBV |
| P014 | Low | Low | 36.5 | 1 | 40.8 | 0 | 57 | Male | F3 | HBV |
| P015 | Low | Low | 38.7 | 0 | 43.1 | 0 | 40 | Female | F1 | HBV |
| P016 | High | High | 41.3 | 1 | 46.2 | 0 | 53 | Male | F2 | HBV |
| P017 | High | High | 43.9 | 1 | 49.5 | 0 | 46 | Male | F2 | HBV |
| P018 | Low | Low | 46.4 | 1 | 52.1 | 1 | 62 | Male | F3 | HBV |
| P019 | High | High | 49.1 | 0 | 55.4 | 0 | 35 | Female | F1 | HBV |
| P020 | Low | Low | 51.8 | 1 | 58.1 | 0 | 54 | Male | F2 | HBV |
| P021 | Low | Low | 54.6 | 0 | 61.3 | 0 | 42 | Female | F2 | HBV |
| P022 | High | High | 57.2 | 1 | 64.5 | 0 | 50 | Male | F2 | HBV |
| P023 | High | High | 60.1 | 0 | 67.2 | 0 | 39 | Male | F1 | HBV |
| P024 | Low | Low | 62.5 | 1 | 70.4 | 1 | 60 | Male | F3 | HBV |
| P025 | High | High | 65.3 | 0 | 73.1 | 0 | 44 | Female | F2 | HBV |
| P026 | Low | Low | 68.7 | 1 | 76.5 | 0 | 56 | Male | F3 | HBV |
| P027 | High | Low | 71.4 | 0 | 79.3 | 0 | 37 | Female | F1 | HBV |
| P028 | Low | High | 74.2 | 1 | 82.4 | 0 | 51 | Male | F2 | HBV |
| P029 | High | High | 77.6 | 0 | 85.7 | 0 | 48 | Female | F2 | HBV |
| P030 | Low | Low | 80.1 | 1 | 88.3 | 1 | 63 | Male | F3 | HBV |
| P031 | Low | High | 83.4 | 0 | 91.2 | 0 | 41 | Male | F1 | HBV |
| P032 | High | Low | 86.2 | 1 | 94.4 | 0 | 58 | Male | F2 | HBV |
| P033 | High | Low | 89.7 | 0 | 97.5 | 0 | 36 | Female | F1 | HBV |
| P034 | Low | High | 92.1 | 1 | 100.2 | 0 | 52 | Male | F2 | HBV |
| P035 | High | High | 95.5 | 0 | 103.8 | 0 | 43 | Female | F2 | HBV |
| P036 | Low | Low | 98.4 | 1 | 106.5 | 1 | 64 | Male | F3 | HBV |
| P037 | High | High | 101.3 | 0 | 109.4 | 0 | 39 | Female | F1 | HBV |
| P038 | Low | Low | 104.6 | 0 | 112.1 | 0 | 55 | Male | F2 | HBV |
| P039 | Low | Low | 107.8 | 0 | 114.8 | 0 | 47 | Female | F2 | HBV |
| P040 | High | High | 110.5 | 0 | 116.9 | 0 | 49 | Male | F2 | HBV |
| P041 | High | High | 113.2 | 0 | 118.4 | 0 | 35 | Female | F1 | HBV |
| P042 | Low | Low | 115.6 | 1 | 119.7 | 1 | 61 | Male | F3 | HBV |
| P043 | High | High | 117.3 | 0 | 120.9 | 0 | 44 | Female | F2 | HBV |
| P044 | Low | Low | 118.9 | 0 | 121.5 | 0 | 53 | Male | F2 | HBV |
| P045 | High | Low | 119.4 | 0 | 122.0 | 0 | 38 | Female | F1 | HBV |
| P046 | Low | High | 120.1 | 0 | 122.4 | 0 | 46 | Male | F2 | HBV |
| P047 | High | High | 120.8 | 0 | 122.8 | 0 | 40 | Female | F1 | HBV |
| P048 | Low | Low | 121.3 | 0 | 123.1 | 0 | 57 | Male | F2 | HBV |
| P049 | High | High | 121.8 | 0 | 123.4 | 0 | 42 | Female | F2 | HBV |
| P050 | Low | Low | 122.2 | 0 | 123.6 | 0 | 56 | Female | F3 | HBV |
| P051 | High | Low | 122.7 | 0 | 123.8 | 0 | 39 | Female | F1 | HBV |
| P052 | Low | High | 113.0 | 0 | 122.8 | 0 | 45 | Male | F2 | HBV |
| P053 | High | High | 118.3 | 0 | 124.0 | 0 | 37 | Female | F1 | HBV |
| P054 | Low | Low | 119.5 | 0 | 123.4 | 0 | 59 | Male | F3 | HBV |
| P055 | High | High | 123.6 | 0 | 123.9 | 0 | 41 | Male | F1 | HBV |
| P056 | Low | Low | 122.9 | 0 | 123.5 | 0 | 54 | Male | F2 | HBV |
| P057 | Low | Low | 120.8 | 0 | 123.7 | 0 | 43 | Female | F2 | HBV |
| P058 | High | High | 123.7 | 0 | 124.0 | 0 | 50 | Male | F2 | HBV |
| P059 | High | High | 124.0 | 0 | 124.0 | 0 | 36 | Female | F1 | HBV |
| P060 | Low | Low | 124.0 | 0 | 123.9 | 0 | 60 | Male | F3 | HBV |
| P061 | High | High | 97.6 | 0 | 111.8 | 0 | 44 | Female | F2 | HBV |
| P062 | Low | Low | 86.9 | 0 | 104.3 | 0 | 58 | Male | F2 | HBV |
| P063 | Low | Low | 45.6 | 1 | 62.8 | 1 | 62 | Male | F3 | HBV |
| P064 | High | High | 103.2 | 0 | 116.9 | 0 | 34 | Female | F1 | HBV |
| P065 | Low | High | 19.8 | 1 | 23.6 | 0 | 58 | Male | F2 | HBV |
| P066 | High | Low | 12.4 | 1 | 31.7 | 0 | 46 | Female | F2 | HBV |
| P067 | Low | Low | 14.7 | 1 | 17.2 | 1 | 59 | Male | F3 | HBV |
| P068 | High | High | 82.5 | 0 | 101.4 | 0 | 39 | Female | F1 | HBV |
| P069 | Low | Low | 19.6 | 1 | 22.8 | 1 | 57 | Male | F3 | HBV |
| P070 | High | High | 76.3 | 0 | 93.8 | 0 | 42 | Female | F2 | HBV |
| P071 | Low | High | 25.7 | 1 | 48.9 | 0 | 52 | Male | F2 | HBV |
| P072 | High | Low | 28.6 | 1 | 32.7 | 1 | 55 | Male | F3 | HBV |
| P073 | Low | Low | 32.9 | 1 | 71.4 | 0 | 60 | Male | F3 | HBV |
| P074 | High | High | 89.7 | 0 | 107.5 | 0 | 37 | Female | F1 | HBV |
| P075 | Low | Low | 39.8 | 1 | 43.5 | 1 | 61 | Male | F3 | HBV |
| P076 | High | High | 91.6 | 0 | 112.3 | 0 | 41 | Female | F1 | HBV |
| P077 | Low | High | 45.9 | 1 | 84.2 | 0 | 49 | Male | F2 | HBV |
| P078 | High | Low | 48.3 | 1 | 88.6 | 0 | 51 | Female | F2 | HBV |
| P079 | Low | Low | 52.7 | 1 | 57.1 | 1 | 58 | Male | F3 | HBV |
| P080 | High | High | 94.8 | 0 | 118.6 | 0 | 38 | Female | F1 | HBV |
| P081 | Low | Low | 59.2 | 1 | 96.5 | 0 | 56 | Male | F2 | HBV |
| P082 | High | High | 101.7 | 0 | 121.4 | 0 | 44 | Female | F2 | HBV |
| P083 | Low | High | 67.4 | 1 | 102.8 | 0 | 53 | Male | F2 | HBV |
| P084 | High | Low | 70.1 | 1 | 75.3 | 1 | 57 | Male | F3 | HBV |
| P085 | Low | Low | 73.6 | 1 | 78.6 | 1 | 60 | Male | F3 | HBV |
| P086 | High | High | 99.4 | 0 | 119.1 | 0 | 35 | Female | F1 | HBV |
| P087 | Low | Low | 79.9 | 1 | 108.4 | 0 | 54 | Male | F2 | HBV |
| P088 | High | High | 106.8 | 0 | 122.7 | 0 | 40 | Female | F1 | HBV |
| P089 | Low | High | 86.7 | 0 | 122.5 | 0 | 48 | Male | F2 | HBV |
| P090 | High | Low | 90.4 | 1 | 115.6 | 0 | 52 | Female | F2 | HBV |
| P091 | Low | Low | 94.8 | 1 | 99.1 | 1 | 59 | Male | F3 | HBV |
| P092 | High | High | 109.6 | 0 | 123.1 | 0 | 43 | Female | F2 | HBV |
| P093 | Low | Low | 102.1 | 1 | 116.6 | 0 | 56 | Male | F2 | HBV |
| P094 | High | High | 110.7 | 0 | 121.9 | 0 | 39 | Female | F1 | HBV |
| P095 | Low | High | 109.5 | 0 | 118.4 | 0 | 51 | Male | F2 | HBV |
| P096 | High | Low | 112.8 | 1 | 116.5 | 1 | 55 | Male | F3 | HBV |
| P097 | Low | Low | 116.2 | 0 | 119.2 | 0 | 58 | Male | F2 | HBV |
| P098 | High | High | 118.4 | 0 | 121.0 | 0 | 36 | Female | F1 | HBV |
| P099 | Low | Low | 120.5 | 0 | 122.5 | 0 | 57 | Male | F3 | HBV |
| P100 | High | High | 121.6 | 0 | 123.2 | 0 | 41 | Female | F2 | HBV |
| P101 | Low | High | 93.6 | 0 | 112.4 | 0 | 49 | Male | F2 | HBV |
| P102 | High | Low | 105.9 | 0 | 120.6 | 0 | 45 | Female | F1 | HBV |
| P103 | Low | Low | 87.8 | 0 | 111.2 | 0 | 60 | Male | F3 | HBV |
| P104 | High | High | 115.3 | 0 | 122.6 | 0 | 38 | Female | F1 | HBV |
| P105 | Low | High | 98.7 | 0 | 117.9 | 0 | 54 | Male | F2 | HBV |
| P106 | High | Low | 119.5 | 0 | 123.4 | 0 | 42 | Female | F2 | HBV |
| P107 | Low | High | 18.6 | 1 | 34.5 | 0 | 47 | Male | F2 | HBV |
| P108 | High | Low | 16.8 | 1 | 19.8 | 1 | 56 | Male | F3 | HBV |
| P109 | Low | Low | 26.9 | 1 | 30.7 | 1 | 61 | Male | F3 | HBV |
| P110 | High | High | 88.6 | 0 | 109.3 | 0 | 35 | Female | F1 | HBV |
| P111 | Low | High | 50.2 | 1 | 86.5 | 0 | 58 | Male | F2 | HBV |
| P112 | High | Low | 96.9 | 0 | 118.2 | 0 | 40 | Female | F1 | HBV |
| P113 | Low | High | 78.3 | 1 | 105.7 | 0 | 53 | Male | F2 | HBV |
| P114 | High | Low | 91.7 | 1 | 96.2 | 1 | 57 | Male | F3 | HBV |
| P115 | Low | High | 105.8 | 1 | 119.7 | 0 | 59 | Male | F3 | HBV |
| P116 | High | Low | 117.8 | 0 | 120.5 | 0 | 37 | Female | F1 | HBV |
| P117 | Low | Low | 108.7 | 0 | 121.6 | 0 | 55 | Male | F2 | HBV |
| P118 | High | High | 113.4 | 0 | 122.9 | 0 | 43 | Female | F2 | HBV |
| P119 | Low | Low | 18.9 | 1 | 52.5 | 0 | 50 | Male | F2 | HBV |
| P120 | High | High | 33.6 | 1 | 37.4 | 1 | 56 | Male | F3 | HBV |
| P121 | Low | Low | 58.7 | 1 | 62.6 | 1 | 62 | Male | F3 | HBV |
| P122 | High | High | 107.2 | 0 | 121.3 | 0 | 39 | Female | F1 | HBV |
| P123 | Low | Low | 113.4 | 0 | 116.8 | 0 | 54 | Male | F2 | HBV |
| P124 | High | High | 121.9 | 0 | 123.6 | 0 | 36 | Female | F1 | HBV |
