# Supplementary material for: Scoparone-Induced Phase Separation of ZDHHC12 Drives Ferroptosis via ATG12 S-palmitoylation and Ferritinophagy in Liver Fibrosis
Source: Research (Wash D C). 2026 Sep 30;9:1450. doi: 10.34133/research.1450 (PMC13624940; doi:10.34133/research.1450)
Supplement: Supplementary file 1 [file research.1450.f1.zip › Table S3.docx]

**Table S3. Clinicopathological characteristics of the liver fibrosis cohort**

| **Patient ID** | **Age (years)** | **Sex** | **Etiology** | **METAVIR Stage** | **ALT (U/L)** | **AST (U/L)** |
| --- | --- | --- | --- | --- | --- | --- |
| P01 | 45 | Male | HBV | F2 | 85.2 | 65.7 |
| P02 | 53 | Male | NAFLD | F3 | 97.1 | 89.3 |
| P03 | 39 | Male | HCV | F1 | 69.3 | 58.1 |
| P04 | 61 | Female | NAFLD | F4 | 154.6 | 114.7 |
| P05 | 48 | Female | HBV | F2 | 88.4 | 69.9 |
| P06 | 56 | Male | HBV | F1 | 68.5 | 57.8 |
| P07 | 42 | Male | AFLD | F3 | 96.6 | 90.2 |
| P08 | 63 | Female | NAFLD | F2 | 87.9 | 70.4 |
| P09 | 50 | Female | HCV | F4 | 157.3 | 148.1 |
| P10 | 46 | Female | AFLD | F1 | 66.7 | 56.5 |
| P11 | 58 | Female | HBV | F3 | 99.2 | 91.7 |
| P12 | 37 | Male | AFLD | F4 | 128.4 | 119.4 |
| P13 | 54 | Male | NAFLD | F4 | 141.2 | 138.6 |
| P14 | 49 | Male | HBV | F2 | 84.7 | 67.3 |
| P15 | 62 | Female | HBV | F1 | 67.3 | 55.2 |
| P16 | 44 | Female | NAFLD | F3 | 94.6 | 90.5 |
| P17 | 53 | Male | HCV | F1 | 62.8 | 57.9 |
| P18 | 41 | Male | NAFLD | F4 | 147.5 | 121.4 |
| P19 | 60 | Female | HBV | F2 | 81.9 | 65.8 |
| P20 | 47 | Female | HBV | F3 | 93.7 | 90.7 |
| P21 | 53 | Male | AFLD | F1 | 81.4 | 56.9 |
| P22 | 59 | Male | NAFLD | F2 | 99.8 | 72.5 |
| P23 | 66 | Male | AFLD | F1 | 82.3 | 57.4 |
| P24 | 64 | Male | HBV | F3 | 125.7 | 98.2 |
| P25 | 61 | Female | HBV | F2 | 102.6 | 69.7 |
| P26 | 69 | Male | NAFLD | F4 | 158.4 | 142.1 |
| P27 | 68 | Female | NAFLD | F1 | 86.5 | 60.3 |
| P28 | 65 | Male | HBV | F2 | 108.2 | 82.1 |
| P29 | 58 | Female | HBV | F4 | 150.7 | 147.4 |
| P30 | 62 | Female | HCV | F3 | 134.6 | 121.5 |
| P31 | 48 | Male | NAFLD | F1 | 78.5 | 56.3 |
| P32 | 52 | Male | AFLD | F1 | 77.2 | 59.8 |
| P33 | 55 | Female | NAFLD | F3 | 125.8 | 109.4 |
| P34 | 57 | Female | AFLD | F2 | 106.9 | 93.7 |
| P35 | 64 | Female | NAFLD | F4 | 152.4 | 146.1 |
| P36 | 62 | Male | AFLD | F3 | 106.3 | 91.5 |
| P37 | 56 | Female | HBV | F4 | 159.7 | 142.6 |
| P38 | 54 | Male | HBV | F4 | 81.5 | 55.9 |
| P39 | 58 | Male | AFLD | F3 | 132.8 | 107.4 |
| P40 | 53 | Female | HBV | F2 | 94.6 | 89.7 |

Abbreviations:

HBV, hepatitis B virus; HCV, hepatitis C virus; NAFLD, non‑alcoholic fatty liver disease; AFLD, alcoholic fatty liver disease; ALT, alanine aminotransferase; AST, aspartate aminotransferase.
